# Correlating Solution‐ and Solid‐State Structures of Conformationally Flexible Resorcinarenes: Significance of a Sulfonyl Group in Intramolecular Self‐Inclusion

**DOI:** 10.1002/chem.201905211

**Published:** 2020-04-30

**Authors:** Małgorzata Pamuła, Maija Nissinen, Kaisa Helttunen

**Affiliations:** ^1^ Department of Chemistry Nanoscience Center University of Jyvaskyla P.O. Box 35 40014 Jyvaskyla Finland

**Keywords:** inclusion compounds, resorcinarenes, solution structures, supramolecular chemistry, X-ray diffraction

## Abstract

The synthesis of tetramethoxyresorcinarene podands bearing *p*‐toluene arms connected by ‐SO_3_‐ (**1**) and ‐CH_2_O‐ (**2**) linkers is presented herein. In the solid state, the resorcinarene podand **1** forms an intramolecular self‐inclusion complex with the pendant *p*‐toluene group of a podand arm, whereas the resorcinarene podand **2** does not show self‐inclusion. The conformations of the flexible resorcinarene podands in solution were investigated by variable‐temperature experiments using 1D and 2D NMR spectroscopic techniques as well as by computational methods, including a conformational search and subsequent DFT optimisation of representative structures. The ^1^H NMR spectra of **1** and **2** at room temperature show a single set of proton signals that are in agreement with *C*_4*v*_ symmetry. At low temperatures, the molecules exist as a mixture of boat conformations featuring slow exchange on the NMR timescale. Energy barriers (Δ*G* ^≠^_298_) of 55.5 and 52.0 kJ mol^−1^ were calculated for the boat‐to‐boat exchange of **1** and **2**, respectively. The results of the ROESY experiments performed at 193 K and computational modelling suggest that in solution the resorcinarene podand **1** adopts a similar conformation to that present in its crystal structure, whereas podand **2** populates a more versatile range of conformations in solution.

## Graphical abstract

**Self‐inclusion**: An intramolecular self‐inclusion complex of a flexible resorcinarene podand stabilised by weak C−H⋅⋅⋅O interactions has been observed in both the crystal structure and in solution (see figure). The solid‐state conformation was determined by XRD and the conformation in solution was investigated by NMR and computational methods, with good agreement observed between the different techniques.

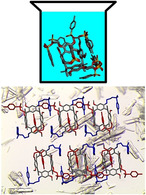

## Introduction

Inclusion complexes, along with the control of self‐inclusion behaviour, play an essential role in the preparation and function of self‐assembled nanomaterials.^1^, ^2^, ^3^ In supramolecular chemistry, macrocyclic hosts are important building blocks providing cavities for inclusion complexation thereby enabling the self‐assembly of multiple supramolecular architectures. These supramolecular assemblies range from simple 1:1 inclusion complexes and pseudorotaxanes^4^, ^5^ to polymers^6^, ^7^, ^8^, ^9^ and particles^10^, ^11^ featuring in some cases gelation properties.^12^ In particular, supramolecular polymers are attracting increasing interest due to their unique properties of self‐healing,^13^ stimuli‐responsiveness^14^ and shape memory.^15^

Intermolecular self‐inclusion is most often detected in crystal structures. Self‐included dimers have been observed in several crystal structures of calixarene macrocycles, such as deep cavitands,^16^ thiacalix[4]arenes^17^ and *N*‐alkyl‐resorcinarene halides.^18^, ^19^ In addition, there are a few examples of crystalline calixarenes featuring chains with head‐to‐tail intermolecular self‐inclusion behaviour.^20^, ^21^ In contrast, intramolecular self‐inclusion requires 180° back‐folding of a part of the compound, typically a covalent host–guest conjugate or a macrocycle equipped with conformationally flexible functional groups. However, crystal structures of intramolecular self‐inclusion monomers are relatively rare.^6^, ^22^, ^23^

The control of intermolecular self‐inclusion is important for the preparation of supramolecular materials and self‐assembled structures. A few examples of pillararenes show a concentration‐dependent transition from intramolecular to intermolecular self‐inclusion in solution, which is utilised for the reversible self‐assembly of dimers or supramolecular polymers.^6^, ^24^ A pillar[5]arene derivative has been reported that provides an interesting example of self‐inclusion complexation in the solid state, with the structure breaking when dissolved in a solvent of small molecular size but remaining intact in a larger‐molecular‐size solvent.^25^ Similarly, a pillar[5]arene‐based pseudo[1]rotaxane that forms a self‐inclusion complex in the solid state shows solvent‐responsive self‐inclusion in solution.^4^ Other examples of self‐inclusion include cyclodextrins, which exhibit self‐inclusion properties when the inclusion of bulky end groups leads to the formation of pseudo[1]rotaxane,^5^ cucurbiturils with a pendant residue acting as hydrophobic guest,^11^ and monosubstituted resorcinarenes^26^, ^27^ and covalently linked dimeric resorcinarene capsules^28^ displaying intramolecular self‐inclusion properties in solution.

Self‐inclusion can also be undesirable for the designed function of a molecular receptor or the self‐assembly of supramolecular aggregates. In the case of a bambusuril anion receptor, solid‐state intramolecular self‐inclusion was observed, and it was expected that this would lower its affinity for guest binding even though self‐inclusion could not be detected in solution.^29^ Intramolecular self‐inclusion of a β‐cyclodextrin was prevented by attaching a bridge across the narrow rim, thus effectively promoting competitive supramolecular polymerisation.^30^

Resorcinarene podands are macrocycles in which the upper‐rim hydroxy groups of the parent resorcinarene have been functionalised, resulting in conformationally flexible octopus‐like structures. In contrast to resorcinarene cavitands with a covalently bridged *C*_4*v*_‐symmetrical crown conformation, resorcinarene podands typically exhibit a *C*_2*v*_‐symmetrical boat conformation in the solid state (CSD version 2019 contained 131 structures identified as *O*‐functionalised resorcinarenes or pyrogallolarenes (podands) and all but one were in the boat conformation). The boat conformation is defined by two resorcinol rings being almost vertically oriented and the two remaining ones horizontally aligned. Previously, self‐inclusion dimers of resorcinarene tetrapodands have been observed by us^31^, ^32^ and others.^33^, ^34^, ^35^, ^36^ Nevertheless, resorcinarene podands tend to display collapsed cavities in the solid state.^37^, ^38^, ^39^, ^40^, ^41^, ^42^, ^43^, ^44^, ^45^

**Scheme 1:**
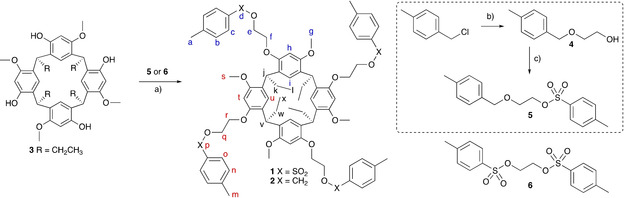
Synthesis of tetramethoxyresorcinarene podands **1** and **2** showing the NMR assignments. For simplicity, only one enantiomer of the tetramethoxyresorcinarene is shown. Reagents and conditions: a) Cs_2_CO_3_, acetonitrile, dibenzo‐18‐crown‐6; b) KOH, ethylene glycol; c) *p*‐toluenesulfonyl chloride, triethylamine, dichloromethane.

**Figure 1:**
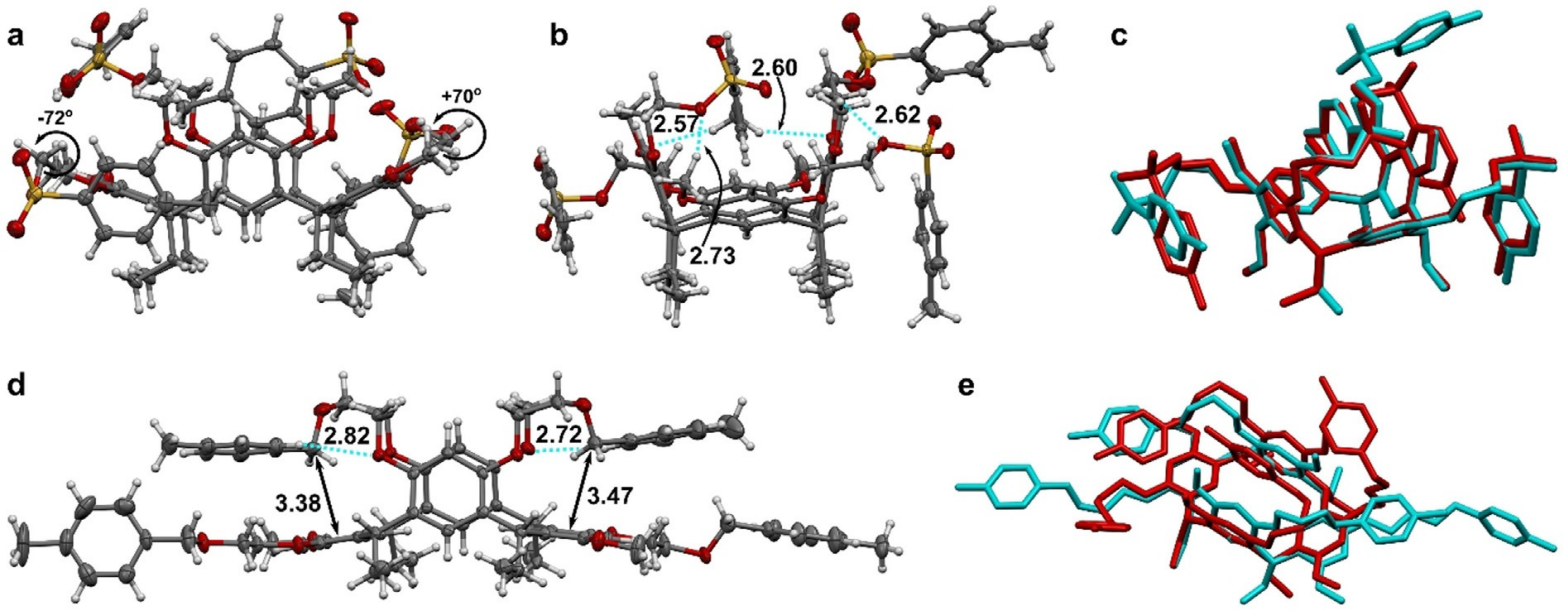
X‐ray structures of a,b) the self‐inclusion complex of resorcinarene podand **1** indicating weak hydrogen bonds (dashed lines) and O‐C‐C‐O dihedral angles, c) an overlay of the X‐ray structure (cyan) and DFT energy‐minimised structure (red) of the lowest‐energy conformation **1**‐I, d) resorcinarene podand **2** showing weak hydrogen bonds (dashed lines) and C−centroid distances (black arrows) and e) an overlay of the X‐ray structure (cyan) and DFT energy‐minimised structure (red) of **2**‐I. Atom colours: C=grey, H=white, S=yellow, O=red. Distances are given in Ångströms.

The crystal structures of conformationally flexible macrocycles lead to the question of how to correlate the solid‐state structure with the conformation of the molecule in solution. Herein, we have addressed this problem by synthesising two resorcinarenes bearing four *p*‐toluene podand arms connected by sulfonyloxy or benzyloxy linkers to provide similar length but different hydrogen‐bond donor/acceptor properties. The single‐crystal X‐ray structure of the sulfonyl‐linked podand **1** (see Figure 1 a,b) reveals an intriguing self‐inclusion complex in which a *p*‐toluene podand arm is included in the resorcinarene cavity. To the best of our knowledge, this is the first example of a resorcinarene podand forming a self‐inclusion complex stabilised only by weak C−H⋅⋅⋅O hydrogen bonding and C−H⋅⋅⋅π interactions. We envision that this structure is rare because it requires inclusion complexation of a pendant *p*‐tolyl group in a non‐preorganised nor permanent aromatic cavity. Indeed, we found only one example of a self‐inclusion complex of a tetramethoxyresorcinarene Schiff base stabilised by NH⋅⋅⋅O hydrogen bonds in the CSD database.^23^ The electron‐rich and ‐poor linker moieties (i.e., sulfonyloxy and ‐CH_2_O‐) included in the podand arms of resorcinarenes **1** and **2**, respectively (see Scheme 1), provide opposite tendencies to weak electrostatic interactions. This allowed us to carry out extensive structural analysis using computational and experimental methods to model the relationship of the crystal structures of **1** and **2** with their conformations in solution. The results show that the solution structure of **1** correlates well with the conformation adopted by the molecule in its crystal structure, whereas **2** populates different types of low‐energy conformations with the possibility of self‐inclusion according to the computational structures.

## Results and Discussion

### Synthesis

Resorcinarene podand **1** was prepared by alkylation of the free hydroxy groups of racemic tetramethoxyresorcinarene (**3**)^46^ with 2‐(tosyloxy)ethyl tosylate (**6**) according to a literature procedure (Scheme 1).^47^ In the podand arm of **1**, a sulfonyl group (‐SO_2_‐) connects the tolyl ring with the 1,2‐dioxyethane chain. To prepare an analogous compound to the resorcinarene podand **1** without the sulfonyl group, resorcinarene podand **2** was synthesised by alkylation of **3** with 2‐[(4‐methylbenzyl)oxy]ethyl tosylate (**5**) in a yield of 44 %. In the podand arm of **2**, the ‐CH_2_O‐ group provides a similar length but lacks the hydrogen‐bond acceptor properties of **1**. The tosylate **5** was prepared in two steps from 4‐methylbenzyl chloride and ethylene glycol. The resorcinarene podands were fully characterised by high‐resolution NMR spectroscopy, ESI‐MS and single‐crystal X‐ray diffraction.

### X‐ray crystallography

Resorcinarene podand **1** was isolated after column chromatographic purification and single crystals suitable for X‐ray diffraction analysis were obtained from ethyl acetate/hexane solution. The crystal structure shows that the resorcinarene macrocycle **1** adopts a boat conformation with two opposite resorcinol rings being almost vertically oriented and the other two horizontally aligned (Figure 1). This conformation is typical for octa‐functionalised resorcinarenes lacking a permanent aromatic cavity. Remarkably, in the case of resorcinarene **1**, the adopted boat conformation defines an aromatic cavity, which is filled by the intramolecular self‐inclusion of one of the terminal tolylsulfonyl substituents of the podand arms. In particular, the self‐inclusion takes place with one of the arms attached to one of the two vertically oriented resorcinol rings (Figure 1). The included tolylsulfonyl group lies at a distance of 3.10–3.45 Å from the vertical resorcinol walls, which indicates the presence of C−H⋅⋅⋅π interactions between them.

The self‐inclusion complex is supported by two intramolecular C−H⋅⋅⋅O bonds formed between the *p*‐methyl group and the resorcinol oxygen atoms of the vertical resorcinol rings (*d*_H⋅⋅⋅O_=2.57–2.60 Å). In addition, the oxygen atoms of the sulfonyloxy group in the included arm reside within a distance of 2.73–2.79 Å from the methoxy and ethoxy protons of one of the two horizontally oriented resorcinol rings.

The two podand arms attached to the horizontal resorcinol rings are wrapped around the resorcinarene cavity. The arms have, however, different O‐C‐C‐O dihedral angles. In one arm, an O‐C‐C‐O torsion angle of +70° induces an intramolecular 2.62 Å contact between a methoxy proton and the sulfonyloxy oxygen on one side (Figure 1 a,b) and rotation of the tolyl group towards the lower rim. On the other side, an O‐C‐C‐O torsion angle of −72° directs the corresponding sulfonyloxy oxygen away from the cavity where it forms weak intermolecular hydrogen bonds. The fourth podand arm, attached to a vertical resorcinol ring, folds to the side of the molecule, engaging in weak intermolecular interactions.

To compare the role of the sulfonyl group in the crystal structure of the podand **1**, single crystals of resorcinarene podand **2** were grown from an ethanol solution. The crystal structure of **2** contains two resorcinarene molecules per asymmetric unit, both adopting a boat conformation. In contrast to resorcinarene **1**, the vertically aligned resorcinol rings lean towards each other, thereby reducing the size of the aromatic cavity of resorcinarene **2** (Figure 1 d). The podand arms attached to the vertical resorcinol rings fold above the horizontal resorcinol rings, placing the benzylic carbon atoms at a distance of 3.38–3.77 Å from the aryl ring centroids. The cavity is sealed by intramolecular C−H⋅⋅⋅O contacts between tolyl ring protons (*ortho* to the CH_2_O substituent) and methoxy oxygen atoms of the vertical resorcinol rings (*d*_H⋅⋅⋅O_=2.65–2.82 Å). The podand arms connected to the horizontal resorcinol rings extend away from the cavity and form edge‐to‐face π⋅⋅⋅π interactions with tolyl arms of adjacent molecules. It should be noted that the second molecule in the asymmetric unit forms a weak intermolecular hydrogen bond between the *p*‐tolyl group attached to the vertical resorcinol ring and a podand arm oxygen of a neighbouring molecule (see the Supporting Information).

### Computational studies

**Figure 2:**
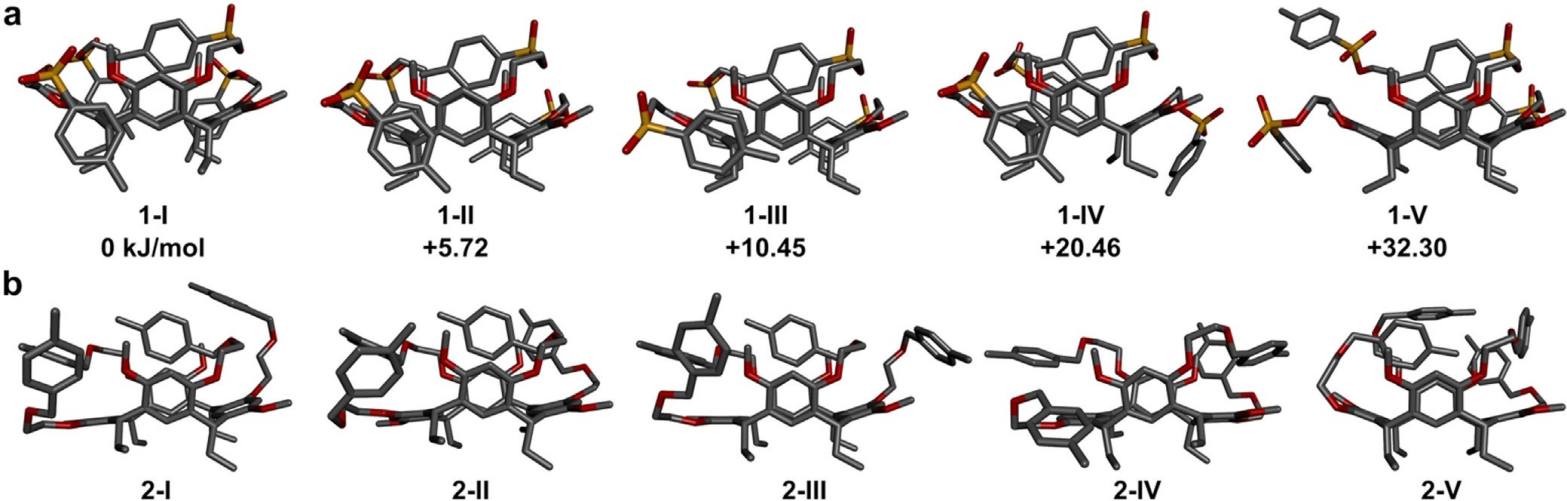
Five representative DFT energy‐minimised conformations a) for **1** with relative DFT B3LYP‐D3 energies and b) for **2**. The relative DFT energies for podand **2** increase in the order **2**‐I<**2**‐V<**2**‐III≈**2**‐II<**2**‐IV.

A conformational search and energy minimisation were performed by means of a molecular mechanics study using the OPLS3e force field and implicit solvent model to generate potential conformations for the resorcinarene podands. The search found 460 potential conformations for podand **1**. In all of these, one vertical arm was folded inside the cavity, similarly to the conformation observed in the crystal structure. The conformations were divided into five categories depending on the folding of the horizontal podand arms and, in particular, the O‐C‐C‐O dihedral angles (Figure 2): in class **1**‐I, both O‐C‐C‐O torsions are positive giving rise to two H_s_⋅⋅⋅O contacts; in class **1**‐II, one O‐C‐C‐O torsion is positive and one is negative resulting in one H_s_⋅⋅⋅O contact; in class **1**‐III, both O‐C‐C‐O torsions are negative; in class **1**‐IV, the horizontal arm proximal to the included vertical sulfonate group does not fold around the cavity; in class **1**‐V, the horizontal arm distal to the included sulfonate group does not fold around the cavity.

The lowest‐energy examples of each class were submitted to DFT geometry optimisation using an implicit solvent model to compare the relative energy differences of each representative conformation (**1**‐I–**1**‐V) at a higher level of theory. The order of increase of the relative energies remained the same as determined in the force‐field energy minimisation (Figure 2 a).

The conformational search for podand **2** found 495 potential conformations. In most of these conformations, a vertical podand arm was back‐folded towards the cavity. These structures were divided into three categories: in class **2**‐I, one horizontal arm is folded on top of the cavity; in class **2**‐II, both horizontal arms are folded at the sides of the cavity; in class **2**‐III, one horizontal arm is back‐folded. In addition, the search found 16 open conformations (class **2**‐IV) without self‐inclusion and seven examples of a horizontal podand arm folding into the cavity (class **2**‐V), which indicates a more versatile range of potential conformations than those found for **1**. Interestingly, the benzylic protons of the vertical aryl rings are directed towards the horizontal resorcinol rings in both the open (**2**‐IV) and self‐inclusion conformations (**2**‐I–**2**‐III). This results in a notable difference in the position and angle of the self‐included tolyl group in **2** compared with in **1**. In **2**, the tolyl group is positioned higher relative to the aromatic cavity and the *p*‐methyl group points upwards, whereas in **1** it points down.

The order of increase of the relative energies of structures **2**‐I–**2**‐V changed after DFT optimisation with the PBE0‐D3 functional, however, structure **2**‐I with self‐inclusion of a vertical podand arm remained as the lowest‐energy conformation. To confirm this result, optimisation was repeated with another popular DFT functional (B3LYP‐D3), which found the same trend in energies but a significant discrepancy in the absolute values along with some structural changes in the optimised geometries (see the Supporting Information). This suggests that the potential energy landscape of podand **2** is more versatile than that of podand **1** containing many local minima.

### ^1^H NMR experiments

**Figure 3:**
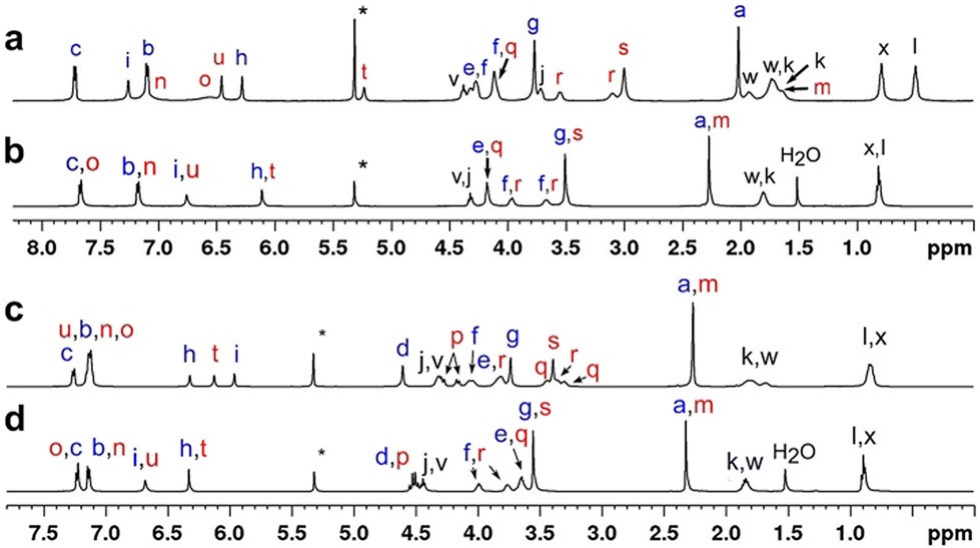
^1^H NMR spectra of the resorcinarene podands in CD_2_Cl_2_: a) **1** at 193 K, b) **1** at 303 K, c) **2** at 193 K and d) **2** at 303 K. Blue letters refer to protons in the horizontally aligned resorcinol rings and red letters to the vertically oriented rings and attached groups.

The conformational characterisation of the resorcinarene podands **1** and **2** in solution was carried out by using NMR spectroscopy. Our aim was to assess how the crystal structures of these conformationally flexible molecules reflect the conformations adopted in solution. The ^1^H NMR spectrum of the tetratolyl‐resorcinarene **2** at 303 K in CD_2_Cl_2_ (Figure 3) displays a single triplet for the methine bridges (H_j,v_), a unique singlet for the methoxy groups (H_g,s_) and two separate singlets for the aromatic protons of the resorcinol rings (H_h,t_ and H_i,u_). The number and multiplicity of the proton signals are in agreement with *C*_4*v*_ symmetry, which probably results from the chemical exchange process between two boat conformations being fast on the ^1^H NMR timescale. The two multiplets observed at *δ*=3.7 and 4.0 ppm, respectively, were assigned to the protons of the methylene group α to the phenol oxygen atom, which resonate as diastereotopic signals (H_f,r_), and the AB quartet at 4.5 ppm was assigned to the ‐CH_2_O‐ methylene protons α to the toluene ring (H_d,p_). In addition to the two singlets of the aromatic resorcinol protons, two sharp doublets corresponding to the protons of the tolyl rings (H_b,n_ and H_c,o_) appear in the aromatic region of the spectrum.

At room temperature, the tetratosylate resorcinarene (**1**) exhibited a similar ^1^H NMR spectrum. The most significant difference is the observation of the methylene protons α to the sulfonyloxy group in **1** appearing as a well‐defined triplet at *δ*=4.18 ppm. The analogous signal for the methylene protons α to the benzyloxy substituent in **2** resonated slightly upfield (*δ*=3.65 ppm) owing to a reduction in the inductive effect of the substituent.

We also recorded the ^1^H NMR spectra of **1** and **2** at different temperatures (303–193 K) to investigate the structural and dynamic properties of the resorcinarene podands at low temperatures, and to determine the energy barriers for the conformational exchange (Figure 3 and the Supporting Information). In addition, the ^1^H NMR spectra of samples with concentrations in the range 1–12 mm were recorded at 193 K to confirm that no dimerisation occurred in this concentration range used for the variable‐temperature experiments.

In the case of **2**, there were no significant changes either in the shape or in the positions of the proton signals when the temperature was lowered to 283 K (see the Supporting Information). As the temperature was further decreased to 263 K, the singlet from the lower‐rim protons (H_i,u_) broadened significantly due to the slowing of the conformational exchange of the two boat conformations, to that close to the NMR timescale. At 243 K, coalescence of the upper rim protons (H_h,t_) and the methoxy groups was observed. Within the temperature range of 223–193 K, new peaks in the aromatic region and at 3.3–4.7 ppm appeared and sharpened, giving rise to three singlets for resorcinol rings (a fourth signal overlaps the tolyl signals at 7.12 ppm, based on ROESY, see the Supporting Information), one signal for methine bridges (H_j,v_) and two singlets for methoxy groups. In addition, a broad doublet with an integral for four protons at 7.26 ppm and a multiplet arising from 12 protons at 7.12 ppm were observed for the tolyl protons H_c_ and H_b_/H_n_/H_o_, respectively. The number and multiplicity of the proton signals at 193 K indicate that the podand adopts a *C*_2*v*_‐symmetrical boat conformation.

The results of the variable‐temperature ^1^H NMR experiments of resorcinarene **1** were very similar to those obtained with **2**. Coalescence was observed at 243 K, which suggests a similar energy barrier to the boat‐to‐boat exchange to that determined for **2**. However, below the coalescence temperature, the lower‐rim methyl signal was split into two broad triplets (H_x_ and H_l_) and the methine bridges appeared as two separate signals (H_v_ and H_j_). Interestingly, half of the tolyl ring protons appeared as sharp doublets at 193 K (H_c_ and H_b_), originating from two magnetically equivalent podand arms, whereas two broad signals were assigned to the tolyl protons (H_n_ and H_o_) of the two remaining podand arms. These broad aromatic signals resonated upfield‐shifted, at 6.60 and 7.10 ppm, relative to their sharply resolved counterparts. Accordingly, the singlet of Ar‐CH_3_ (H_a_) had an integral equivalent of six protons, and the remaining Ar‐CH_3_ protons (H_m_) were most likely located together with the lower‐rim CH_2_ signals H_w_ and H_k_ based on the integral values of these broad resonances.

The line shapes of the NMR signals arising from the lower‐rim CH_3_ protons (H_l_ and H_x_) and upper‐rim resorcinol protons (H_h_ and H_t_) at different temperatures were modelled by line‐shape analysis to obtain the exchange rate constants (*k*). The exchange rate constants were analysed by means of Eyring plots (see the Supporting Information), which provided Gibbs free energies of activation (Δ*G* ^≠^_298_ ) of 55.5 kJ mol^−1^ for **1** and 52.0 kJ mol^−1^ for **2** (Table 1). These values are similar to the value obtained for octa‐acetylated resorcinarene podands (Δ*G* ^≠^_298_=54.7 kJ mol^−1^).^35^

**Table 1:** Activation energy parameters for the conformational exchange in compounds **1** and **2**.

Molecule	Δ*G* ^≠^_298_ [kJ mol^−1^]	Δ*H* ^≠^ [kJ mol^−1^]	Δ*S* ^≠^ [J mol^−1^ K^−1^]
**1**	55.5	21.0	−116.0
**2**	52.0	28.0	−80.7

### ^1^H–^1^H COSY and ROESY NMR spectroscopy

**Figure 4:**
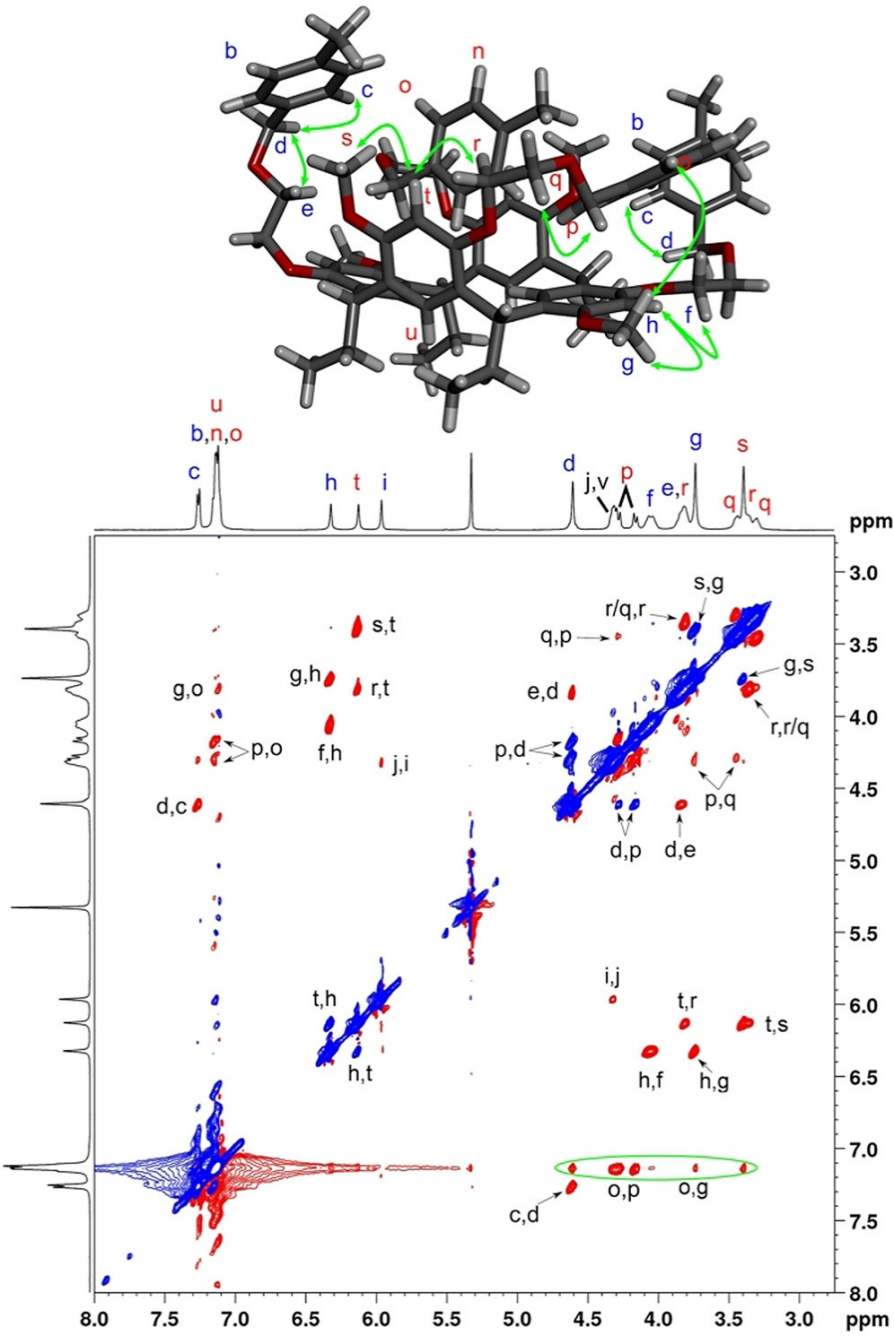
^1^H–^1^H ROESY spectrum of **2** recorded at 193 K in CD_2_Cl_2_ and DFT‐minimised structure **2**‐I showing the key contacts (<4 Å distance) present in this conformation matching the ROESY data. Negative ROE cross‐peaks are shown in red and positive exchange cross‐peaks in blue. The overlap of b, n, o and u protons hinder the assignment of ROEs in the green circle.

^1^H–^1^H COSY and ROESY NMR spectroscopy were used to investigate the conformations of the resorcinarene podands **1** and **2** in solution, as well as to confirm the assignment of the protons at low temperature. The ROESY measurements of **2** at 193 K showed strong ROE correlations between aryl protons and methoxy protons, ArH_h_ to H_g_ and ArH_t_ to H_s_, as well as between aryl and ethoxy protons, ArH_h_ to H_f_ and ArH_t_ to H_r_ (Figure 4), which indicates that these protons are coupled through space. On the other hand, ROE correlations were observed for aryl protons ArH_i_ with methine H_j_/H_v_, which allowed the assignment of H_i_ as one of the lower‐rim protons and ArH_h_ and ArH_t_ as the upper‐rim protons of the resorcinol rings.

Positive exchange correlations were also observed between H_d_ and H_p_, which were assigned to benzylic protons. The signal H_d_ resonated as an AB quartet with almost identical chemical shifts of the A and B protons, whereas Δ*δ*_AB_ for H_p_ was 0.12 ppm. The upfield shift in signal H_p_ in comparison with signal H_d_ suggests that the proton is magnetically shielded by the proximity of an aromatic ring. This condition is met for the podand arm attached to the vertical resorcinol ring in the conformation observed in the X‐ray structure, in which the benzylic protons establish C−H⋅⋅⋅π interactions with the horizontally aligned resorcinol rings. Based on the computational models, the shielding of the protons H_p_ may result from the inclusion of one of the podand arms in the resorcinarene cavity and from the above‐mentioned C−H⋅⋅⋅π interactions with a resorcinol ring. Thus, the protons H_d_–H_i_ arise from the horizontally aligned resorcinol ring in a boat conformation, and the protons H_p_–H_u_ were assigned to the vertically opposed resorcinol ring.

**Figure 5:**
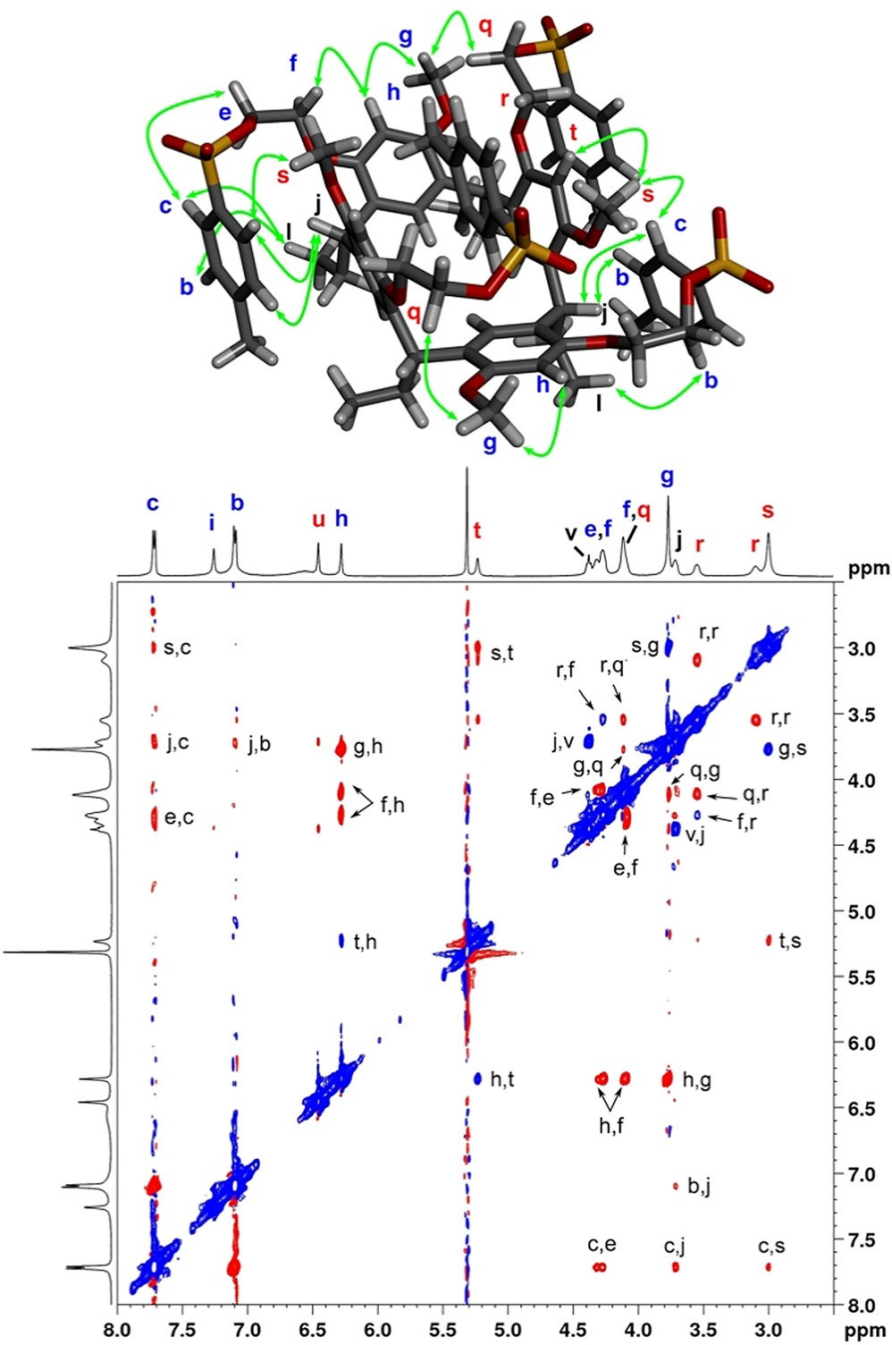
^1^H–^1^H ROESY of **1** recorded at 193 K in CD_2_Cl_2_ and DFT‐minimised structure of **1**‐I showing the key contacts (<4 Å distance) present in this conformation matching the ROESY data. Negative ROE cross‐peaks are shown in red and positive exchange cross‐peaks in blue.

The ROESY spectrum of **1** at 193 K (Figure 5) revealed a similar pattern of exchange cross‐peaks between methoxy protons H_g_ and H_s_, and the upper‐rim protons ArH_h_ and ArH_t_ as in the spectrum of **2**. Interestingly, the methoxy group H_g_ showed a ROE correlation with the protons assigned to H_f_/H_q_, which was not observed in **2**. Comparison of the DFT energy‐minimised structure and the X‐ray structure showed that from these two possibilities the distance between H_g_ and H_q_ (horizontal–vertical, ca. 3 Å) is significantly shorter than the distance between H_g_ and H_f_ (horizontal–horizontal, ca. 4 Å), and therefore a more likely source of this correlation. This assignment was made assuming that the similar chemical shifts of the methoxy groups in **1** and **2** allow the assignment of H_g_ and H_s_ to horizontally and vertically oriented resorcinol rings also in **1**.

ROE correlations from the sharp tolyl group signals H_c_ and H_b_ were observed to the resorcinol lower‐rim groups. Proton H_b_ showed weak correlations with methine H_j_ and methyl H_l_, and proton H_c_ showed cross‐peaks with the same lower‐rim protons and, interestingly, to the ethoxy H_e_/H_f_ and methoxy H_s_. This combination of ROEs is best explained if the tolyl group bearing H_c_ and H_b_ is attached to the horizontal resorcinol ring and wraps around the aromatic cavity thereby approaching the vertical methoxy group H_s_ and the lower rim of the podand. This interpretation is in agreement with our tentative assignment of the vertically and horizontally aligned resorcinol rings as well as with the crystal structure of **1**.

The computational structures **1**‐I–**1**‐V are in excellent agreement with the observed ROEs. In particular, structure **1**‐I, which also has the lowest DFT energy, shows all the expected distances from the horizontal podand arm protons H_b_ and H_c_ to the lower‐rim protons and the vertical methoxy protons H_s_. In the structures **1‐**IV and **1**‐V, one of the horizontal arms folds under the horizontally aligned resorcinol ring. In this conformation, the distances between the tolyl group protons and lower‐rim protons are too long for the observed ROEs, which suggests that these conformations are less populated in solution, in accordance with their higher DFT energies.

It is important to note that the tolyl groups of the two podand arms attached to the vertical resorcinol rings show broad H_m_, H_n_ and H_o_ signals at 193 K and appear upfield‐shifted relative to the corresponding H_a_, H_b_ and H_c_ signals in the horizontal arm. This indicates that the tolyl groups are engaged in a dynamic process that is intermediate on the NMR chemical shift timescale. Based on the most likely conformation of molecule **1** in solution (ROEs and DFT structures), especially the proximity of the vertical podand arm ethoxy protons H_q_ to the horizontal ring protons H_g_, it can be deduced that the conformation of the vertical podand arm is favourable for tolyl group inclusion into the aromatic resorcinol cavity. The upfield‐shifted and broadened tolyl group signals are indicative of a second exchange process with a low energy barrier involving dynamic inclusion of the tolyl groups in the magnetically shielded environment of the resorcinarene cavity.

## Conclusions

We have presented the synthesis of two conformationally flexible resorcinarene podands without permanent cavities that were characterised by a complete set of high‐resolution spectroscopic techniques and X‐ray crystallography. In the solid state, the resorcinarenes exhibit different conformations of the *p*‐toluene podand arms, with the sulfonyloxy linker in **1** promoting self‐inclusion complexation of the *p*‐toluene group and the benzyloxy linker in **2** pinching of the resorcinarene cavity. Variable‐temperature NMR experiments revealed an energetic barrier in the range of 52–55 kJ mol^−1^ for the boat‐to‐boat exchange for both podands, which indicates that the different podand arm linkers do not influence significantly the exchange rate.

The conformations and intramolecular interactions of the macrocycles were also investigated by ROESY experiments at low temperature. Furthermore, a conformational search and DFT optimisations were carried out to explore the conformation space of the podands. The observed ROE correlations between the podand arms and resorcinol protons suggest that the tetratosylate resorcinarene podand **1** exhibits a similar wrapped conformation of the horizontal podand arms in solution as observed in the solid state. Furthermore, the magnetic shielding of the broad aromatic tolyl group signals assigned to the vertical podand arms and the computational modelling results suggest that the self‐inclusion of the *p*‐toluene group is a highly favourable conformation also in solution.

For the tetratolyl‐resorcinarene **2**, the ^1^H NMR and ROESY data suggest that the benzylic protons of the vertical podand arms are involved in C−H⋅⋅⋅π interactions with the horizontally aligned resorcinol rings in solution, which was also observed in all the computational structures. Computational modelling revealed a more versatile conformation space for resorcinarene **2** than for **1**, for which self‐inclusion of a vertical or horizontal podand arm was identified as the lowest‐energy structure. However, the conformational search also found an open conformation without self‐inclusion, which deviates from the crystal structure most significantly by a more compact orientation of the horizontal podand arms. Therefore, it is expected that both types of conformations are populated in solution, whereas the extended conformation of the horizontal podand arms is an effect induced by the favourable π⋅⋅⋅π interactions in crystal packing.

The role of the sulfonyloxy linker in the self‐inclusion complex of resorcinarene **1** is two‐fold. On the one hand, the sulfonyloxy oxygen atoms are suitable hydrogen‐bond acceptors for establishing weak hydrogen bonds that support the self‐inclusion complex, and on the other hand, the oxygen lone pairs direct the podand arms away from the horizontally aligned resorcinol rings. Comparison with resorcinarene **2** clearly indicates that the presence of the sulfonyl group restricts the range of conformations the podand is likely to adopt. Ultimately, we can conclude that the conformation of podand **1** in solution correlates well with its solid‐state structure, whereas the conformation of podand **2** changes between self‐inclusion complexes and open conformations.

## Experimental Section

Solvents and reagents were purchased from the commercial suppliers Sigma–Aldrich and VWR. Solvents were dried by using an MBraun solvent purification system. The syntheses were performed with dried glassware (at 120 °C overnight) under a balloon of nitrogen. Flash chromatography was performed with a Combiflash Companion (Teledyne Isco) and Redisep Gold silica columns. Mass spectrometry was performed with a Micromass LCT ESI‐TOF or an ABSciex QSTAR Elite ESI‐Q‐TOF mass spectrometer. Melting points were measured with a Stuart Scientific SMP30 instrument and are uncorrected.

NMR spectra were recorded with a Bruker Avance III 500 spectrometer (500 MHz for ^1^H and 126 MHz for ^13^C). Chemical shifts (*δ*) are given in ppm and coupling constants (*J*) are given in Hz. Variable‐temperature NMR experiments of the podands were performed with a BBI probe. The ^1^H NMR spectra were recorded in CD_2_Cl_2_ at different temperatures (303, 283, 263, 243, 223, 203 and 193 K) at a concentration of 9 mm for **1** and 11 mm for **2**. ROESY and COSY ^1^H–^1^H NMR experiments were performed at 193 K. Complete line‐shape analysis was performed with Bruker TopSpin software (TopSpin 3.5, Bruker BioSpin Corporation, 2017).

**Syntheses**

**Resorcinarene 1**: Compound **1** was synthesised as described previously.^47^ ^1^H NMR (500 MHz, CD_2_Cl_2_, +30 °C): *δ*_H_=7.67 (d, *J=*8.2 Hz, 8 H; H_c_, H_o_), 7.18 (d, *J=*8.0 Hz, 8 H; H_b_, H_n_), 6.76 (s, 4 H; H_i_, H_u_), 6.11 (s, 4 H; H_h_, H_t_), 4.32 (t, *J=*7.9 Hz, 4 H; H_j_, H_v_), 4.17 (t, *J=*4.8 Hz, 8 H; H_e_, H_q_), 4.01–3.93 (m, 4 H; H_f_, H_r_), 3.73–3.62 (m, 4 H; H_f_, H_r_), 3.51 (s, 12 H; H_g_, H_s_), 2.27 (s, 12 H; H_a_, H_m_), 1.87–1.73 (m, 8 H; H_w_, H_k_), 0.82 ppm (t, *J=*6.7 Hz, 12 H; H_l_, H_x_); ^13^C NMR (126 MHz, CD_2_Cl_2_, +30 °C): *δ*_C_=156.15, 155.29, 145.65, 133.63, 130.41, 128.29, 127.35, 126.67, 126.45, 98.26, 69.42, 67.54, 55.77, 36.62, 28.82, 21.69, 12.78 ppm.

**Resorcinarene 2**: Tetramethoxyresorcinarene **3** (0.200 g, 0.304 mmol), Cs_2_CO_3_ (0.804 g, 2.468 mmol) and dibenzo‐18‐crown‐6 (0.010 g, 0.028 mmol) were mixed with dry acetonitrile (30 mL) under a nitrogen atmosphere at 100 °C with stirring. A white suspension formed. The mixture was heated at reflux for 15 min before the addition of **5** (0.428 g, 1.338 mmol) in dry acetonitrile (20 mL). The resulting suspension was then heated at reflux for 24 h. The solution was filtered while hot through a pad of Celite, and the solid residue was washed with hot acetonitrile. The solvent was evaporated on a rotary evaporator, and the residue was dissolved in dichloromethane (50 mL) and washed with water (20 mL). The organic solution was dried with MgSO_4_ and filtered, evaporated and purified by flash chromatography on a SiO_2_ column with a hexane/ethyl acetate gradient (from 8:2 to 0:1). The product was recovered as a colourless oil and recrystallised from ethanol as a white crystalline solid (yield: 44 %). M.p. 92–93 °C; ^1^H NMR (500 MHz, CD_2_Cl_2_): *δ*_H_=7.24 (d, *J=*7.9 Hz, 8 H; H_c_, H_o_), 7.14 (d, *J=*7.9 Hz, 8 H; H_b_, H_n_), 6.68 (s, 4 H; H_i_, H_u_), 6.33 (s, 4 H; H_h_, H_t_), 4.51 (ABq, Δ*δ*_AB_=0.04, *J*_AB_=11.5 Hz, 4 H; H_d_, H_p_), 4.44 (t, *J*=7.2 Hz, 4 H; H_j_, H_v_), 4.05–3.93 (m, 4 H; H_f_, H_r_), 3.81–3.71 (m, 4 H; H_f_, H_r_), 3.71–3.59 (m, 8 H; H_e_, H_q_), 3.55 (s, 12 H; H_g_, H_s_), 2.32 (s, 12 H; H_a_, H_m_), 1.93–1.75 (m, 8 H; H_w_, H_k_), 0.89 ppm (t, *J*=7.2 Hz, 12 H; H_l_, H_x_); ^13^C NMR (126 MHz, CD_2_Cl_2_): *δ*_C_=156.12, 155.67, 137.50, 135.90, 129.17, 128.00, 126.35, 126.28, 98.24, 73.49, 69.54, 69.40, 55.79, 37.22, 28.12, 21.10, 12.85 ppm; HRMS (ESI‐TOF): *m*/*z* calcd for C_80_H_96_O_12_Na^+^: 1271.6794 [*M*+Na^+^]; found: 1271.6785.

**Compound 4**: KOH (0.41 g, 7.37 mmol) was dissolved in anhydrous ethylene glycol by heating to 90–100 °C under a nitrogen atmosphere. 4‐Methylbenzyl chloride (1.00 g, 7.11 mmol) was added dropwise to the yellow solution and heating was continued at 90 °C for 24 h. The yellow mixture was diluted with Millipore water (30 mL) and extracted with dichloromethane (3×20 mL). The combined extracts were washed once with water and dried with MgSO_4_ and filtered. The organic solvent was evaporated and the residue was purified by flash chromatography using a SiO_2_ column and a petroleum ether/ethyl acetate gradient (from 65:35 to 0:1). The product was isolated as a colourless liquid (yield: 0.63 g, 53 %) and used as such in the next step. ^1^H NMR (500 MHz, CDCl_3_, +30 °C): *δ*_H_=7.24 (d, *J=*8.0 Hz, 2 H), 7.17 (d, *J=*8.0 Hz, 2 H), 4.52 (s, 2 H), 3.77–3.72 (m, 2 H), 3.60–3.56 (m, 2 H), 2.35 ppm (s, 3 H); ^13^C NMR (126 MHz, CDCl_3_): *δ*_C_=137.5, 134.9, 129.1, 127.9, 73.1, 71.1, 61.9, 21.1 ppm.

**Compound 5**: Tosyl chloride (0.768 g, 4.028 mmol) was dissolved in dry dichloromethane (30 mL) under a nitrogen atmosphere, and the solution was cooled in an ice bath. Triethylamine (0.56 mL, 4.015 mmol) was added, followed by 2‐*O*‐(4‐methylbenzyl)ethyl alcohol (**4**; 0.56 g, 3.363 mmol) in dichloromethane (15 mL). The mixture was then stirred for 1 h in the ice bath and for 24 h at room temperature. The resulting brown solution was washed with water (2×40 mL). The resulting yellowish organic solution was dried with MgSO_4_ and filtered, and evaporated to dryness. The product was purified by flash chromatography using a SiO_2_ column and a hexane/ethyl acetate gradient (from 8:2 to 0:1). The product was recovered as a colourless liquid (yield: 0.494 g, 46 %) with traces of ethyl acetate and used as such in the next step. ^1^H NMR (500 MHz, CDCl_3_, 25 °C): *δ*_H_=7.79 (d, *J=*8.1 Hz, 2 H; Ts‐*H*), 7.31 (d, *J=*8.1 Hz, 2 H; Ts‐*H*), 7.14 (br s, 4 H; Ar‐*H*), 4.44 (br s, 2 H; Ar‐C*H*_2_), 4.18 (t, *J=*4.7 Hz, 2 H; SO_3_‐C*H*_2_), 3.63 (t, *J=*4.7 Hz, 2 H; O‐C*H*_2_), 2.43 (s, 3 H; Ts‐C*H*_3_), 2.34 ppm (s, 3 H; Ar‐C*H*_3_); ^13^C NMR (126 MHz, CDCl_3_): *δ*_C_=144.9, 137.7, 134.6, 133.2, 129.9, 129.2, 128.1, 127.9, 73.3, 69.4, 67.4, 21.8, 21.3 ppm; MS (ESI‐TOF): *m*/*z*=343.6 (100) [*M*+Na]^+^.

**X‐ray crystallography**: Single‐crystal X‐ray diffraction data were recorded on an Agilent SuperNova, single source at offset, Eos diffractometer using an Agilent Atlas CCD detector with mirror monochromatised Mo_Kα_ irradiation (*λ*=0.71073 Å). The data were processed and empirical absorption correction was made by using CrysAlisPro (CrysAlisPro 1.171.38.41, Rigaku Oxford Diffraction, 2015). The structures were solved with the Superflip (L. Palatinus, G. Chapuis, *J. Appl. Crystallogr*. **2007**, *40*, 786–790) structure solution program using Charge Flipping (L. Palatinus, *Acta Crystallogr. Sect. B Struct. Sci. Cryst. Eng. Mater*. **2013**, *69*, 1–16) or a SHELXS (G. M. Sheldrick, *Acta Crystallogr. Sect. A Found. Crystallogr*. **2008**, *64*, 112–122) structure solution program using direct methods. Structure refinement was carried out with Olex2 (O. V Dolomanov, L. J. Bourhis, R. J. Gildea, J. A. K. Howard, H. Puschmann, *J. Appl. Cryst*. **2009**, *42*, 339–341) using the SHELXL (G. M. Sheldrick, *Acta Crystallogr. Sect. A Found. Crystallogr*. **2008**, *64*, 112–122) refinement package with least‐squares minimisation. Hydrogen atoms were calculated in their idealised positions with isotropic temperature factors (1.2 or 1.5 times the C temperature factor) and refined as riding atoms. EADP and EXYZ constraints, and SADI, DFIX, DELU and SIMU restraints were used in the refinement of the disordered fragments in **2**. The crystallographic parameters are shown in the Supporting Information.

CCDC 1963708 (**1**) and 1963709 (**2**) contain the supplementary crystallographic data for this paper. These data are provided free of charge by The Cambridge Crystallographic Data Centre.

**Computational study**: A conformational search was performed for individual molecules of **1** and **2** using the Maestro package^48^ in a mixed torsional low mode sampling mode with 10 000 steps. Crystal coordinates were used as the starting point and energy minimisation of the structures was performed with molecular mechanics using the OPLS3e force field. The resulting structures were classified in relevant conformations and the lowest‐energy example of each representative conformation was submitted to DFT structure optimisation using the Terachem software.^49^, ^50^ DFT calculations were performed by using the two popular functionals B3LYP‐D3^51^, ^52^ and PBE0‐D3^53^ with dispersion correction,^54^, ^55^ the 6‐31G** basis set and the PCM solvent model^56^ (parameters for chloroform: dielectric constant=4.806 and probe radius=2.52 Å).

## Conflict of interest

The authors declare no conflict of interest.

## Supporting information

As a service to our authors and readers, this journal provides supporting information supplied by the authors. Such materials are peer reviewed and may be re‐organized for online delivery, but are not copy‐edited or typeset. Technical support issues arising from supporting information (other than missing files) should be addressed to the authors.

Supplementary
