# Supplementary material for: Correlating Solution‐ and Solid‐State Structures of Conformationally Flexible Resorcinarenes: Significance of a Sulfonyl Group in Intramolecular Self‐Inclusion
Source: Chemistry. 2020 Apr 30;26(33):7374–83. doi: 10.1002/chem.201905211 (PMC7317440; doi:10.1002/chem.201905211)
Supplement: Supplementary file 1 — Supplementary [file CHEM-26-7374-s001.pdf]

Chemistry–A European Journal

Supporting Information

Correlating Solution- and Solid-State Structures of Conformationally Flexible Resorcinarenes: Significance of a Sulfonyl Group in Intramolecular Self-Inclusion

Małgorzata Pamuła, Maija Nissinen, and Kaisa Helttunen*[a]

Contents:

NMR spectra of the compounds 2

Compound 4 2

Compound 5 4

Resorcinarene 1 6

Resorcinarene 2 7

VT NMR experiments 8

Complete lineshape analysis 12

2D NMR experiments 15

Resorcinarene 1 15

Resorcinarene 2 18

X-ray crystallography 22

Computational results 24

NMR spectra of the compounds

Compound 4

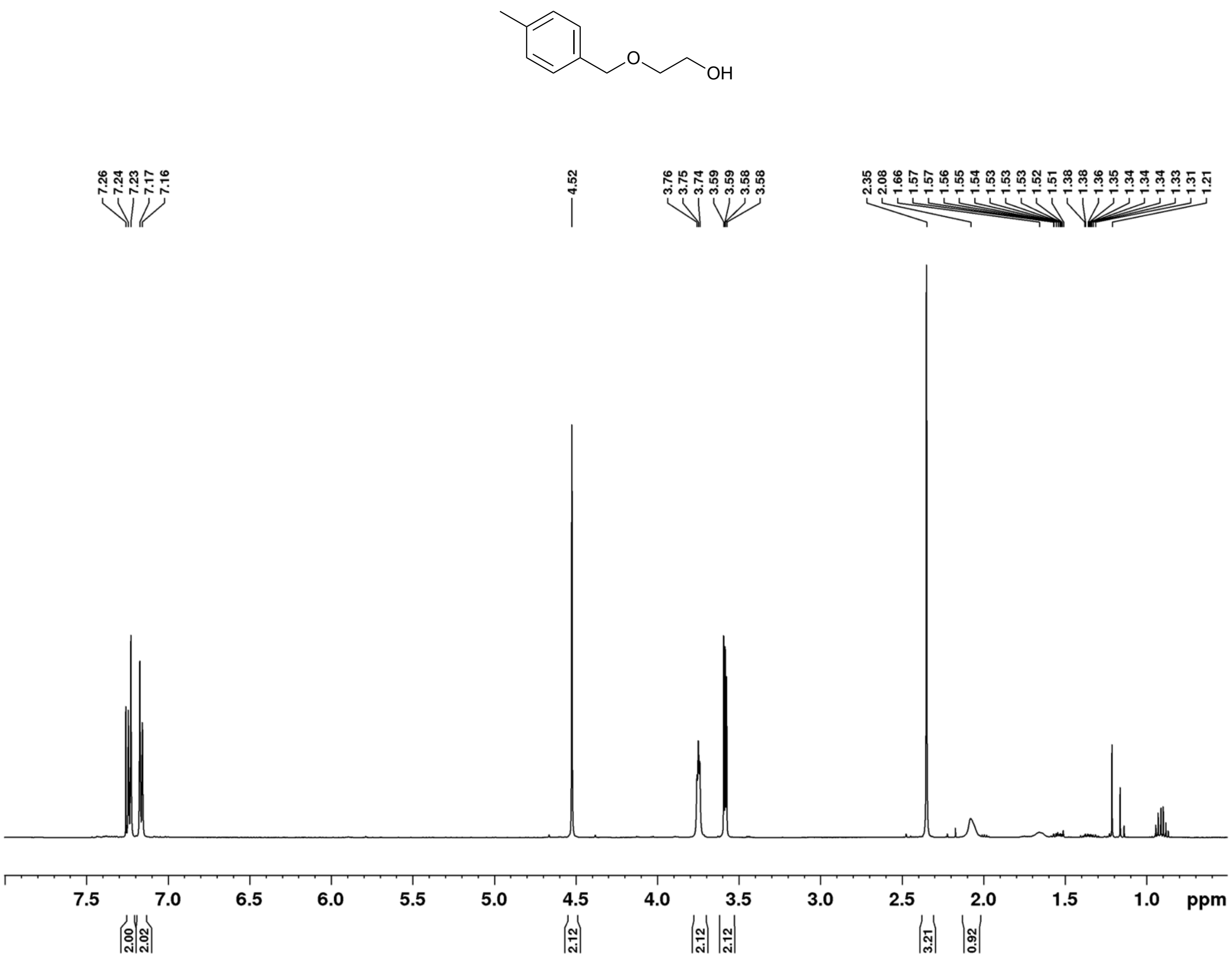

Fig. S-1 ^{1}H NMR spectrum of **4** in $CDCl_3$ (25 °C, 500 MHz).

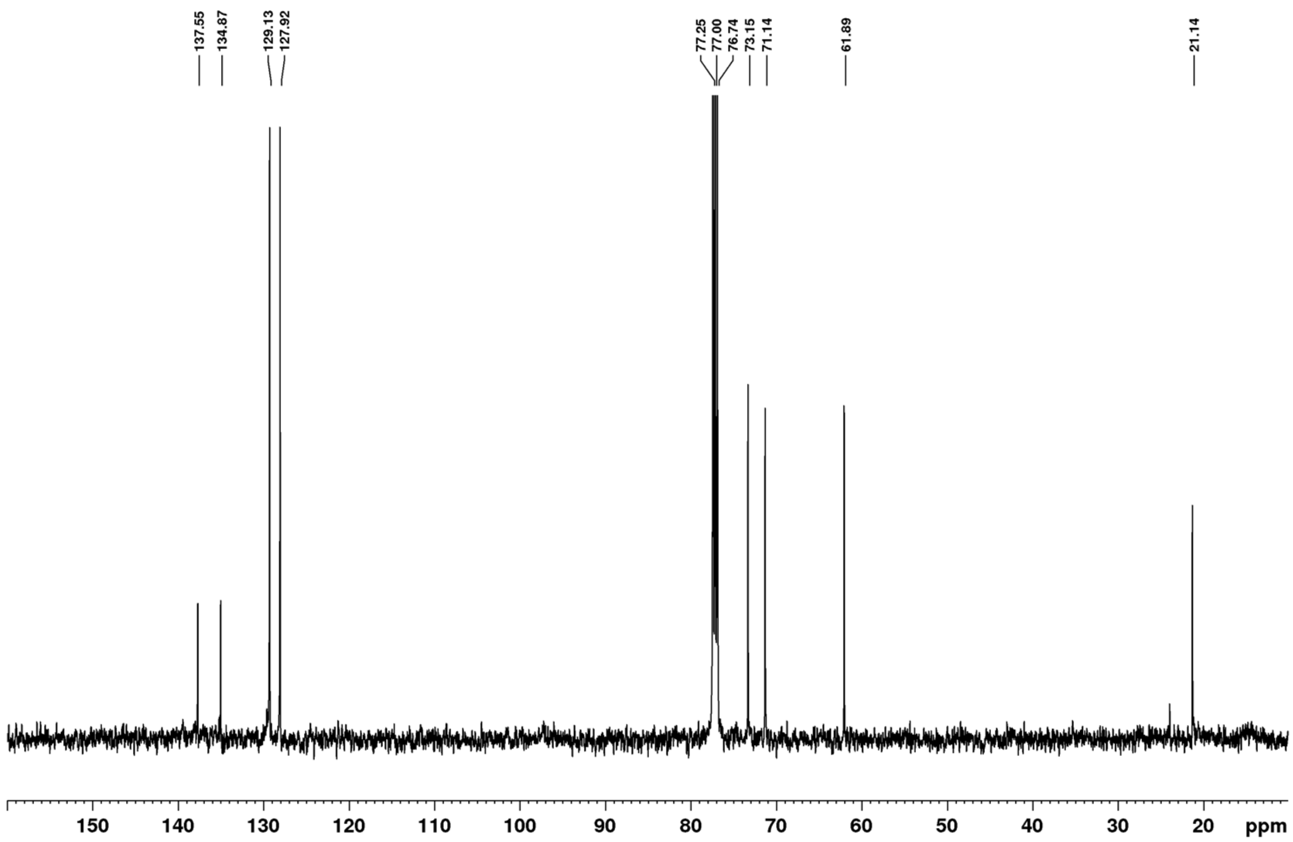

Fig. S-2 ^{13}C NMR spectrum of **4** in $CDCl_3$ (25 °C, 500 MHz).

Compound 5

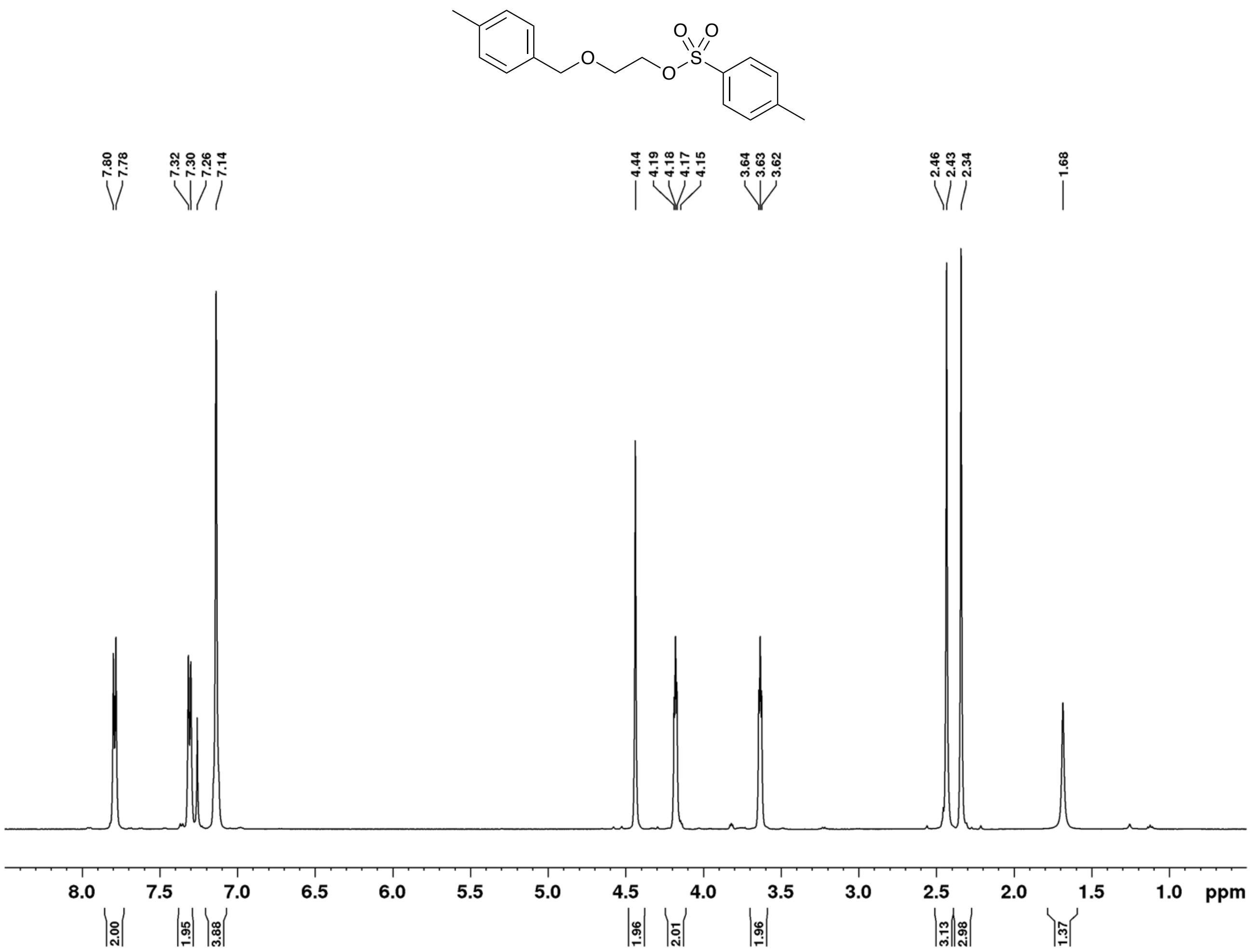

Fig. S-3 ^{1}H NMR spectrum of **5** in $CDCl_3$ (25 °C, 500 MHz).

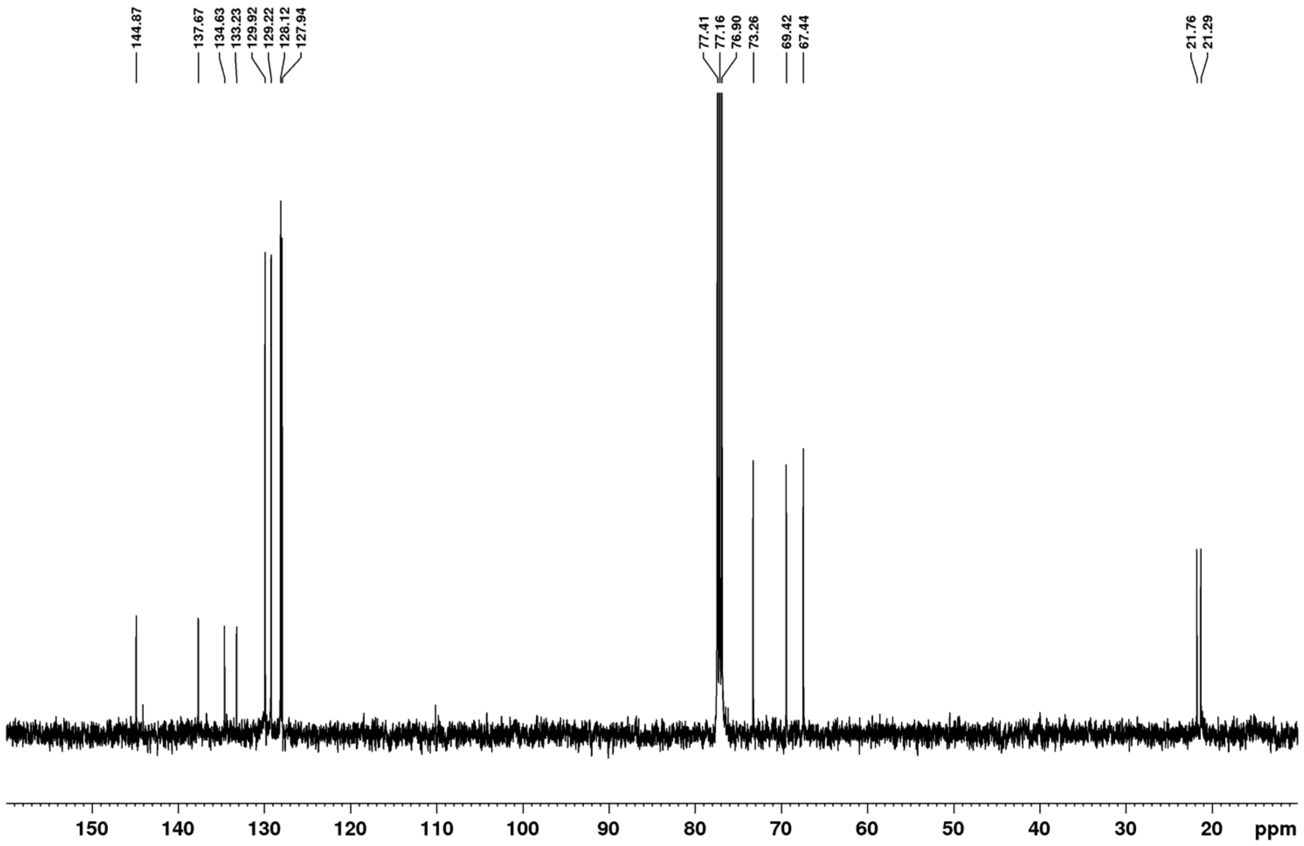

Fig. S-4 ^{13}C NMR spectrum of **5** in $CDCl_3$ (25 °C, 126 MHz).

Resorcinarene 1

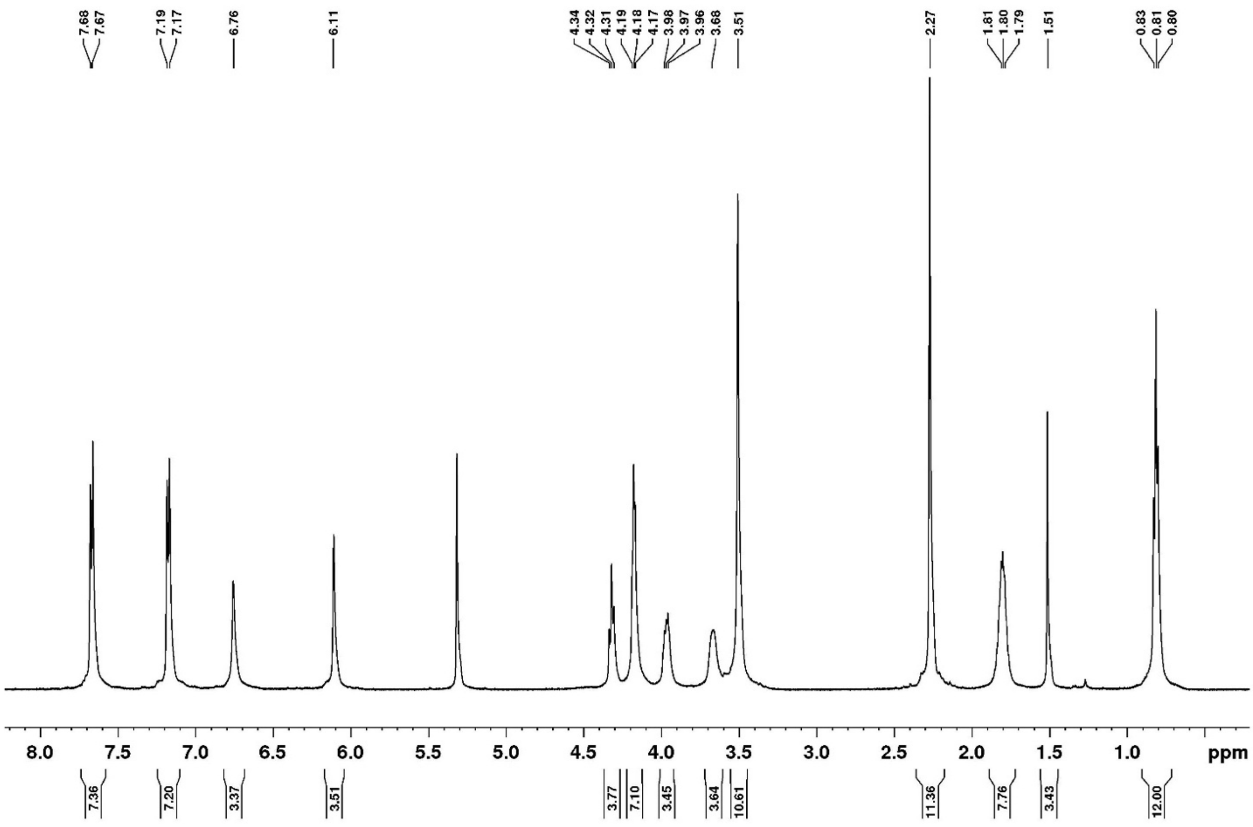

Fig. S-5 ^{1}H NMR spectrum of **1** in CD_2Cl_2 (30 °C, 500 MHz).

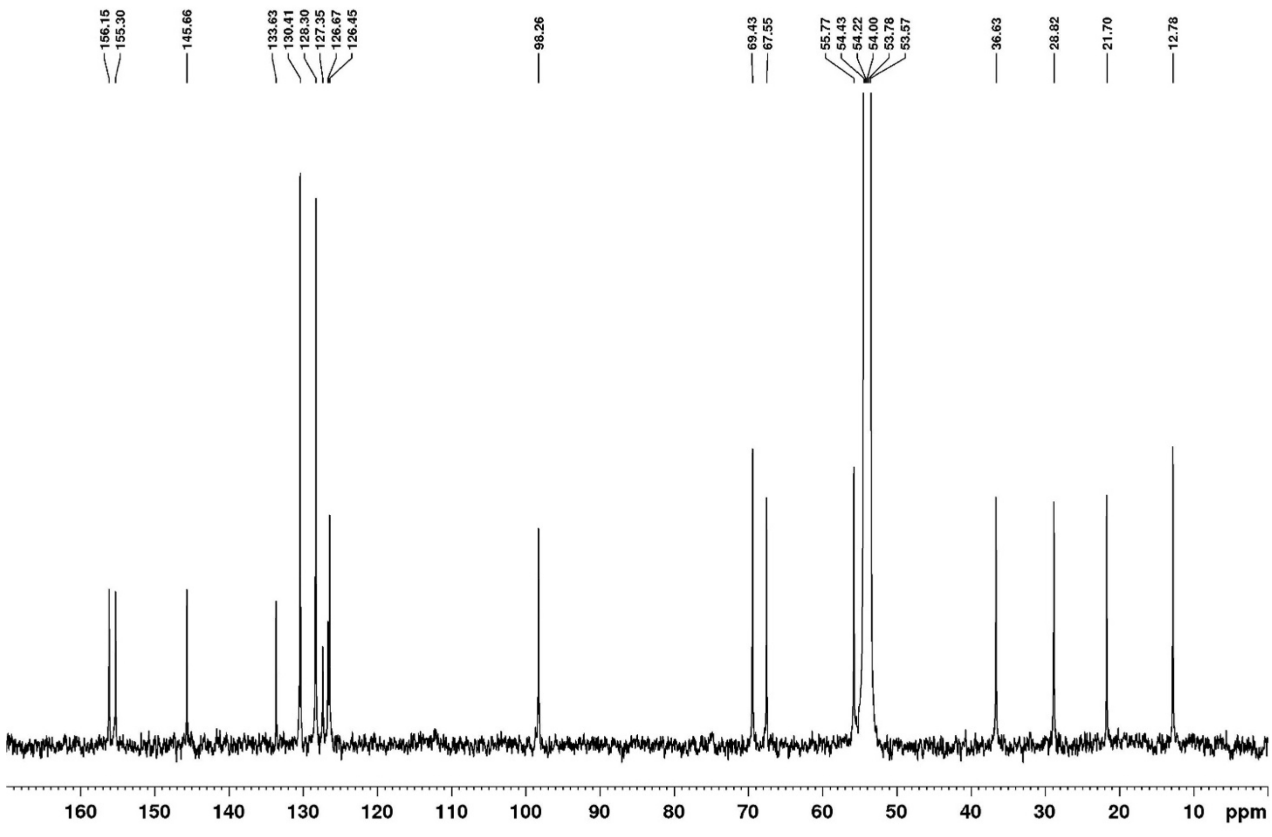

Fig. S-6 ^{13}C NMR spectrum of **1** in CD_2Cl_2 (30 °C, 500 MHz).

Resorcinarene 2

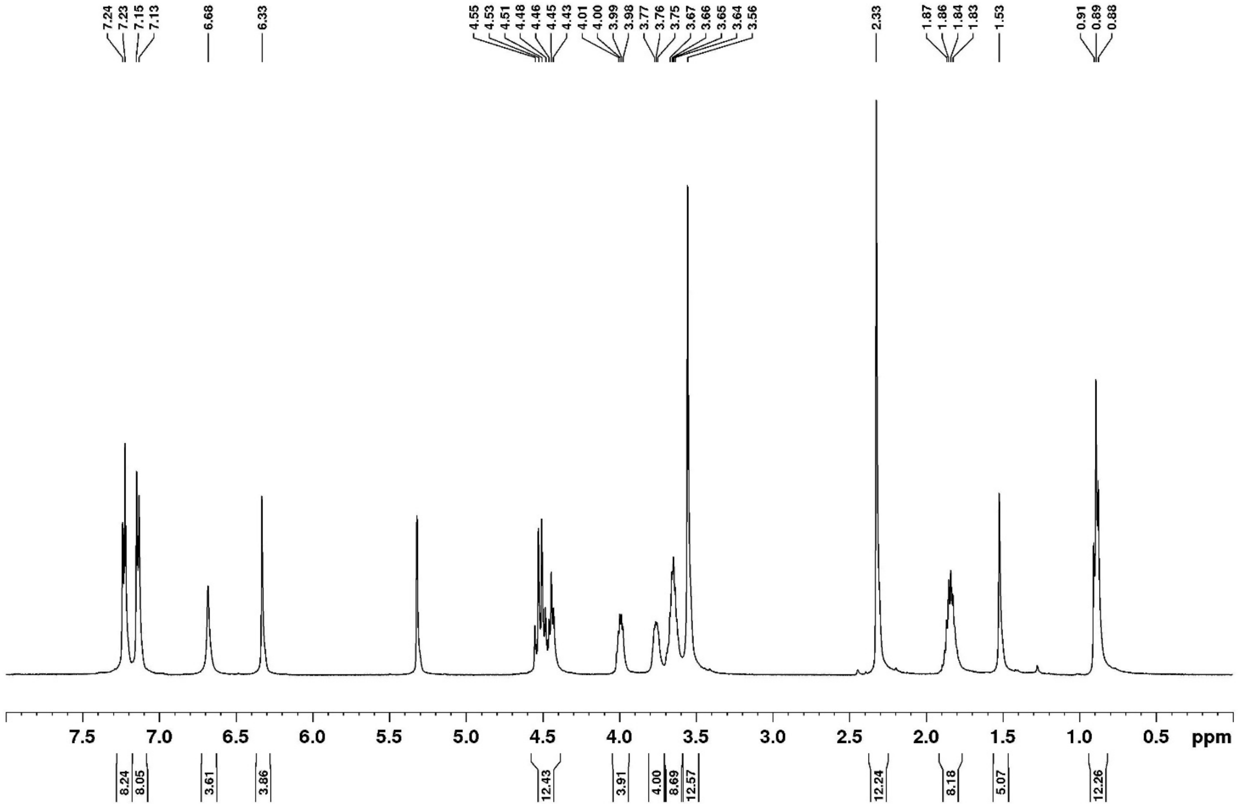

Fig. S-7 ^{1}H NMR spectrum of **2** in CD_2Cl_2 (30 °C, 500 MHz).

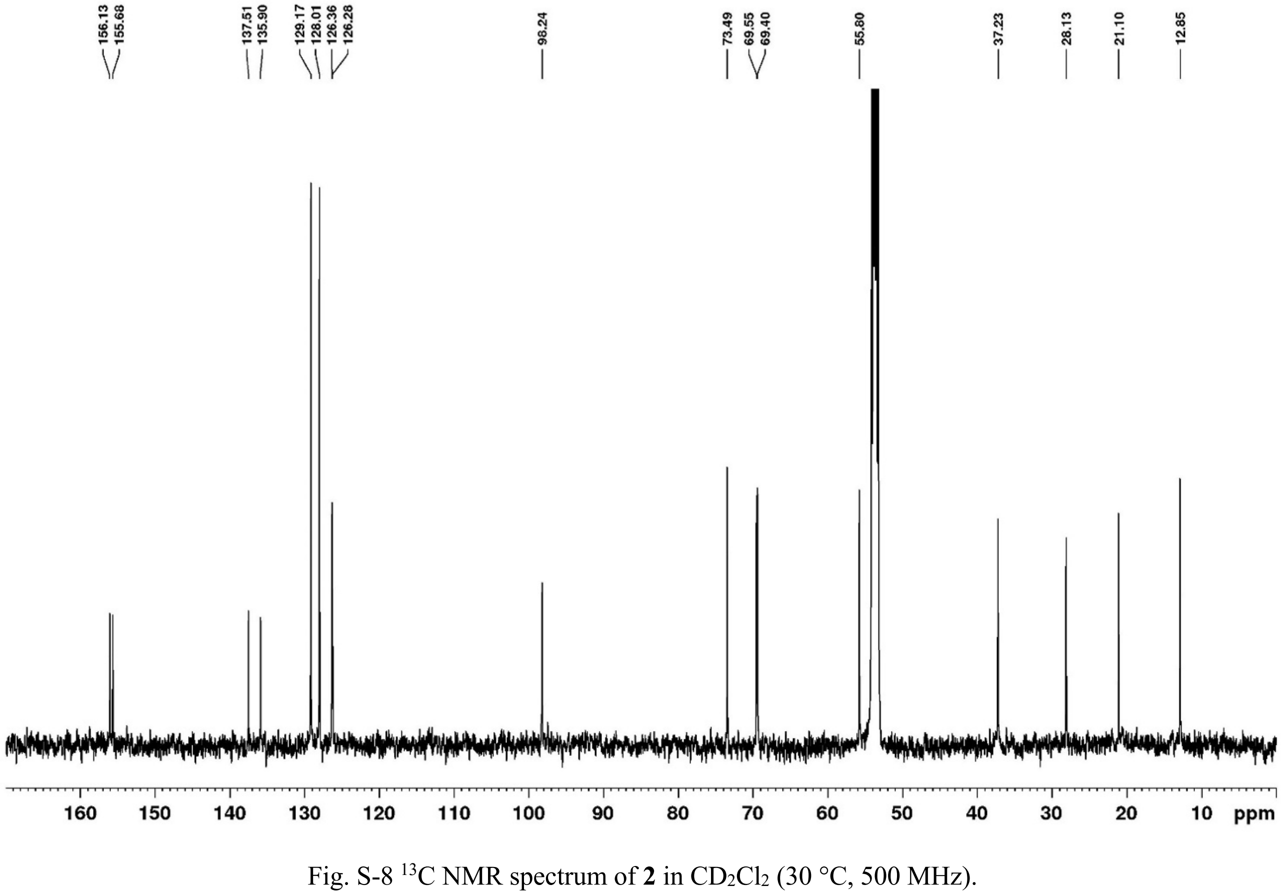

Fig. S-8 ^{13}C NMR spectrum of **2** in CD_2Cl_2 (30 °C, 500 MHz).

VT NMR experiments

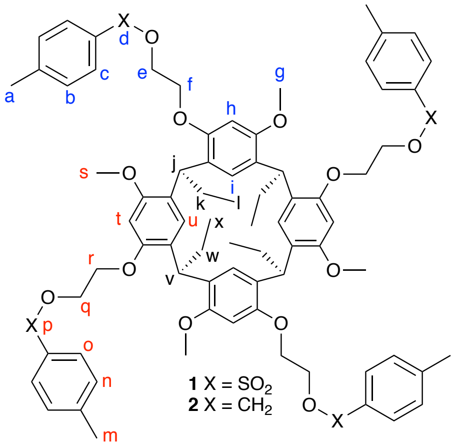

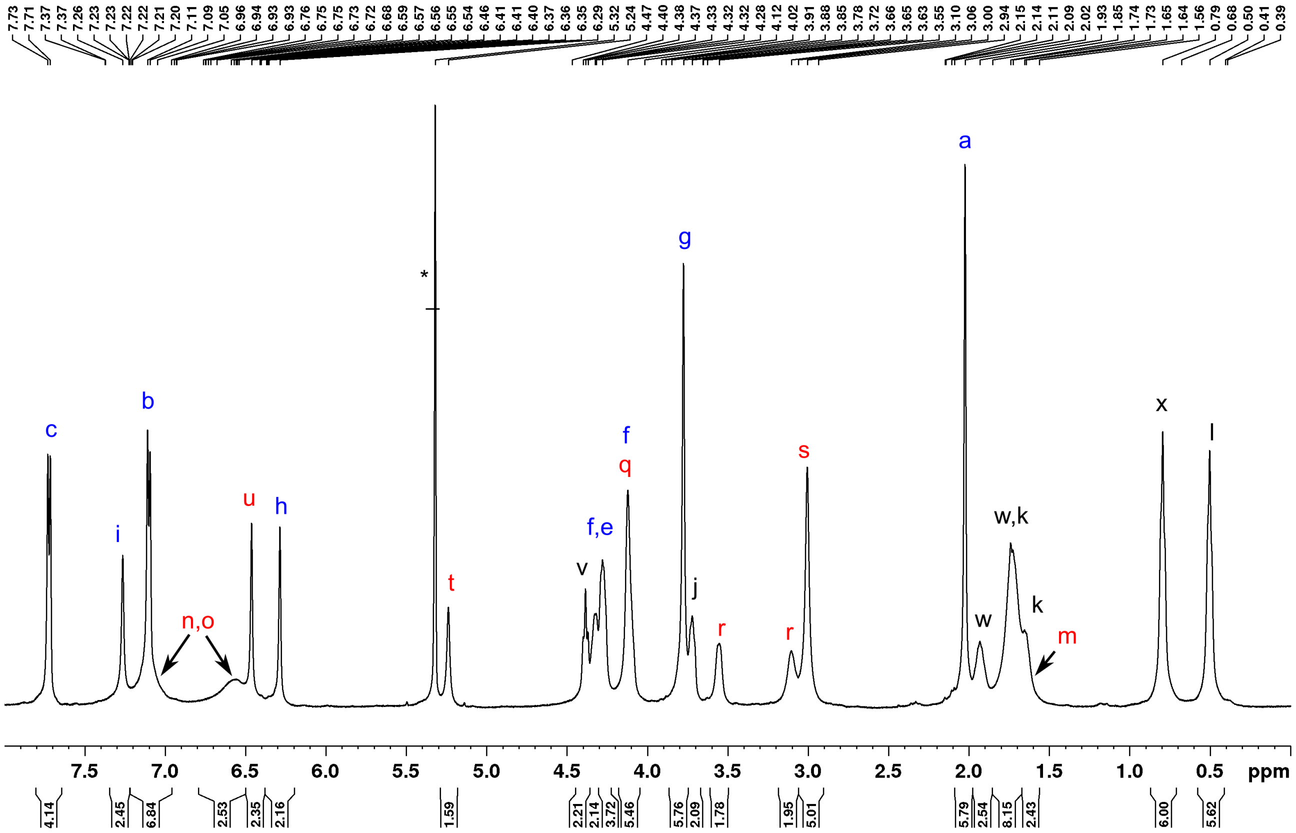

Fig. S-9 ^{1}H NMR spectrum of **1** in CD_2Cl_2 (-80°C, 500 MHz). Solvent marked with an asterisk. Blue letters refer to the horizontally aligned resorcinol ring and red letters to the vertically aligned ring and attached groups.

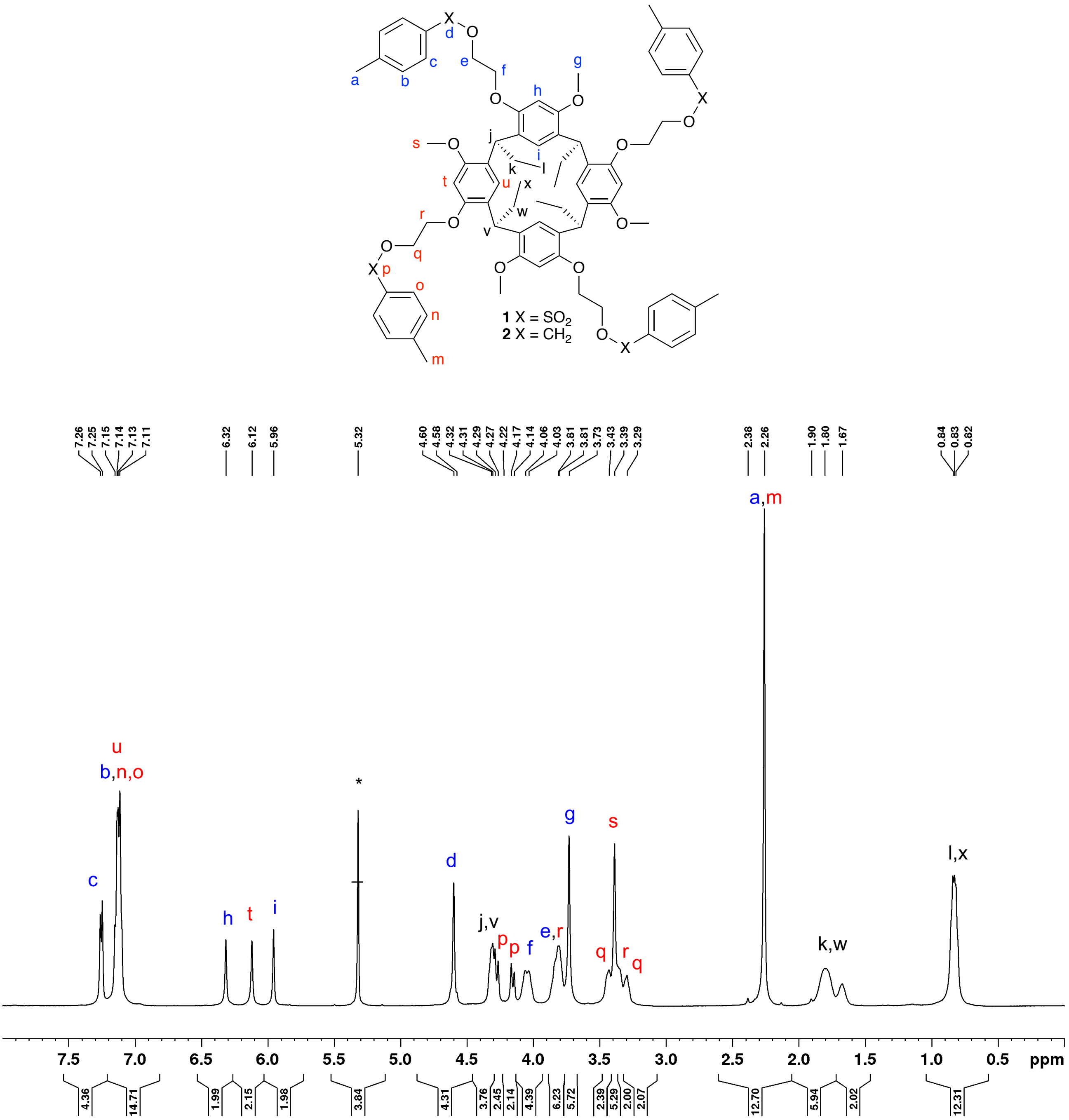

Fig. S-10 ^{1}H NMR spectrum of **2** in CD_2Cl_2 (-80°C, 500 MHz). Solvent marked with an asterisk. Blue letters refer to the horizontally aligned resorcinol ring and red letters to the vertically aligned ring and attached groups.

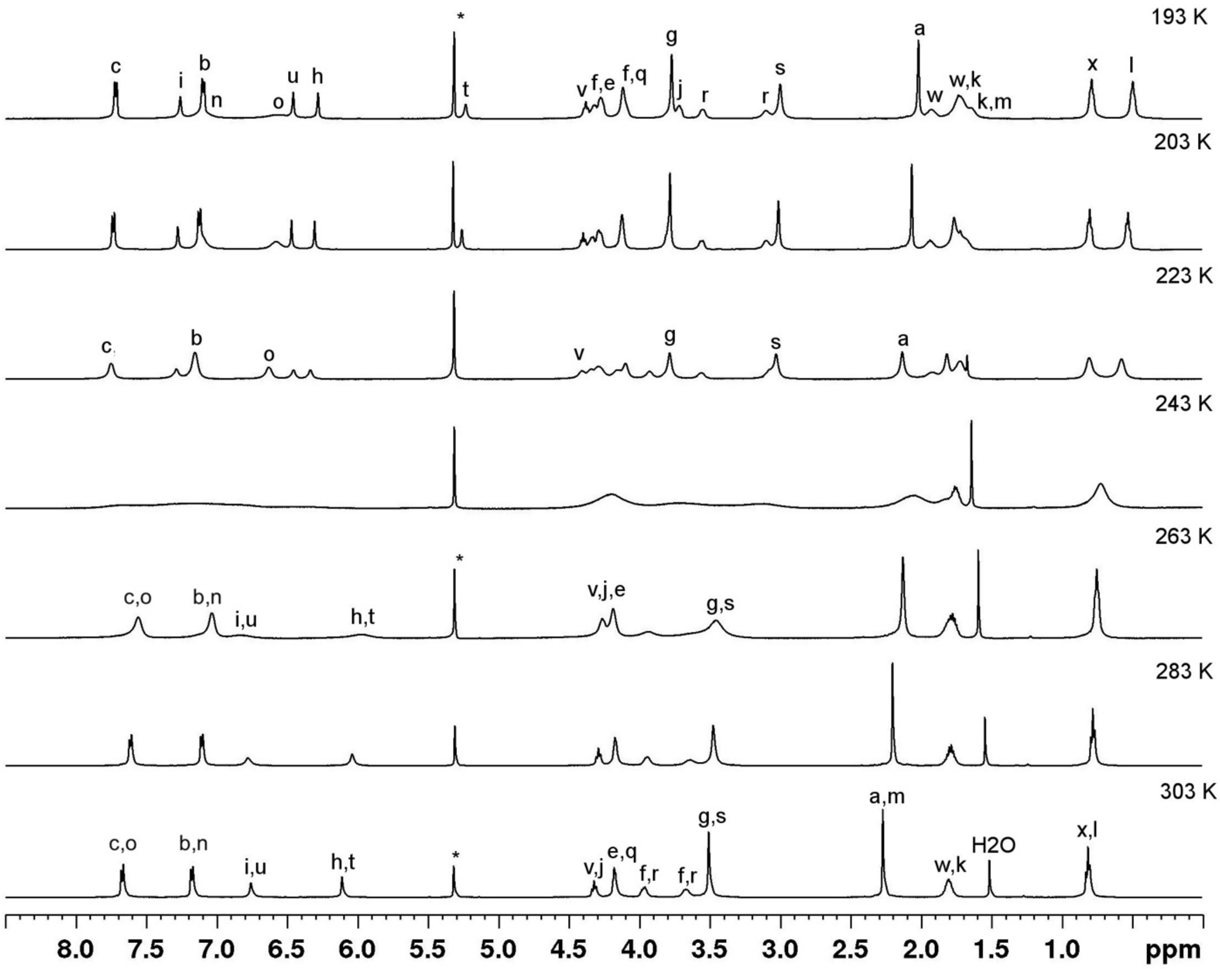

Fig. S-11 VT ^{1}H NMR spectrum of **1** in CD_2Cl_2 (-80°C, 500 MHz).

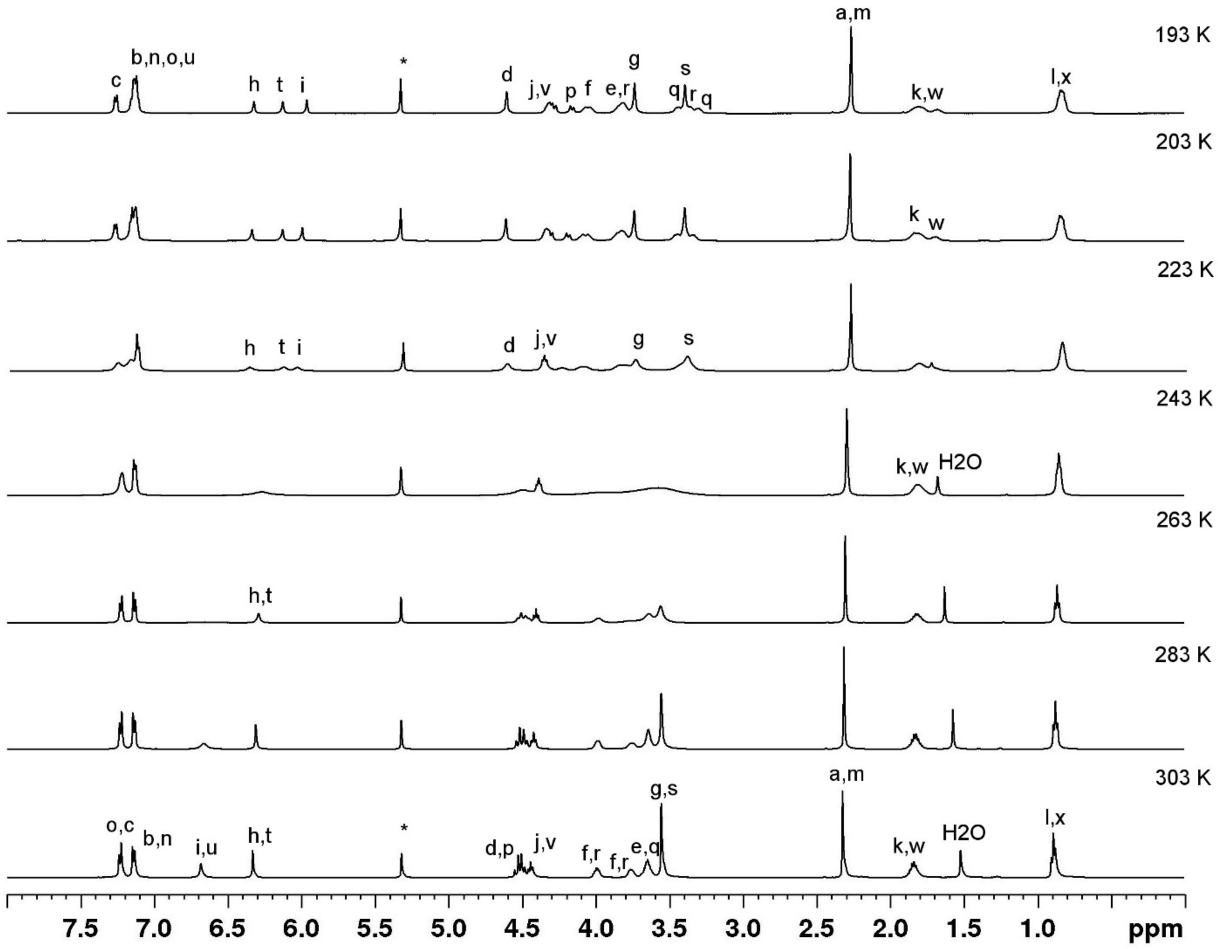

Fig. S-12 VT ^{1}H NMR spectrum of **2** in CD_2Cl_2 (-80°C, 500 MHz).

Complete lineshape analysis

Exchange rates (k) were obtained from complete lineshape analysis using TopSpin v. 3.5. The fitting was performed using line broadening (LB) parameter of 4 Hz.

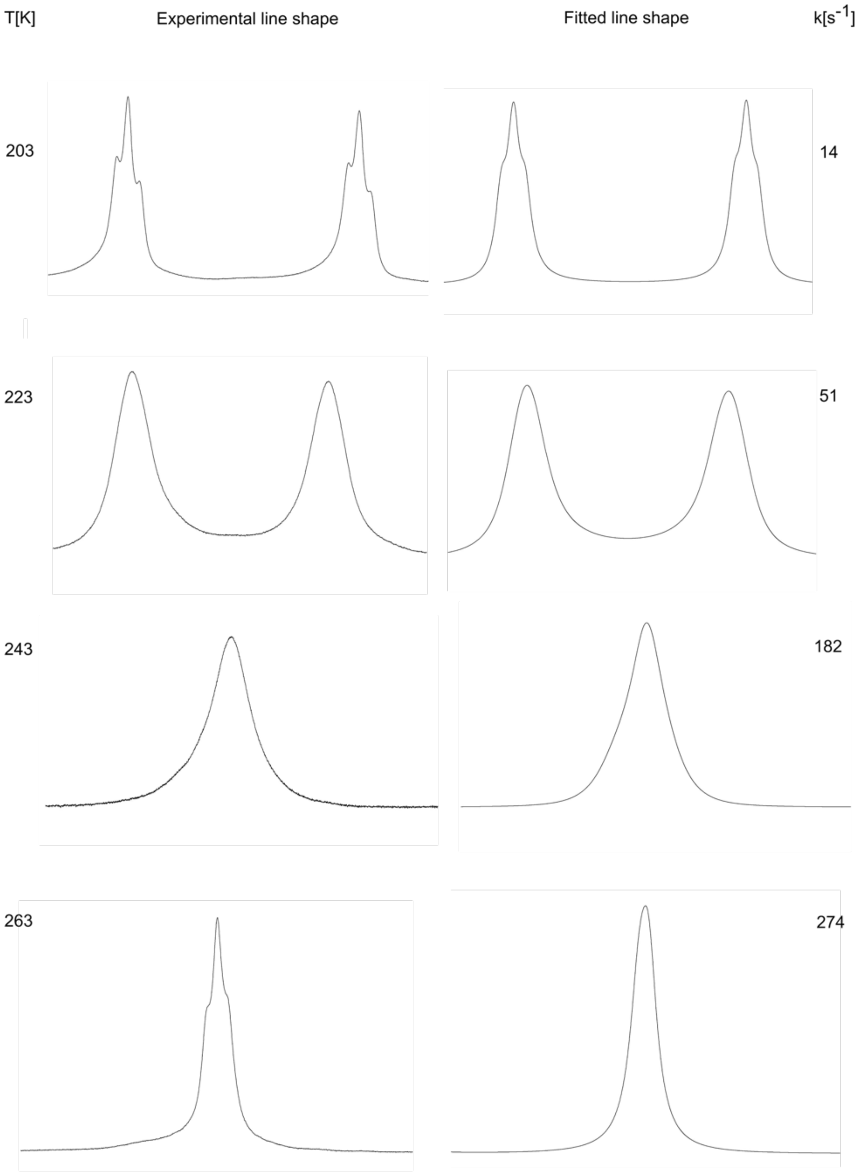

Fig. S-13 Complete line shape fitting results for methyl protons H_l and H_x, which were used for the Eyring plot of **1**.

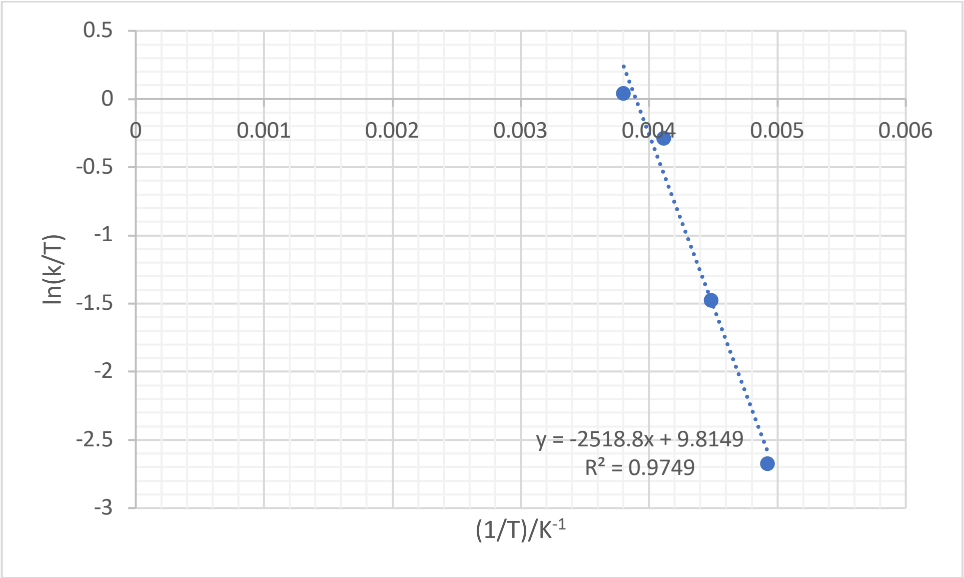

Fig. S-14 Eyring plot of **1**.

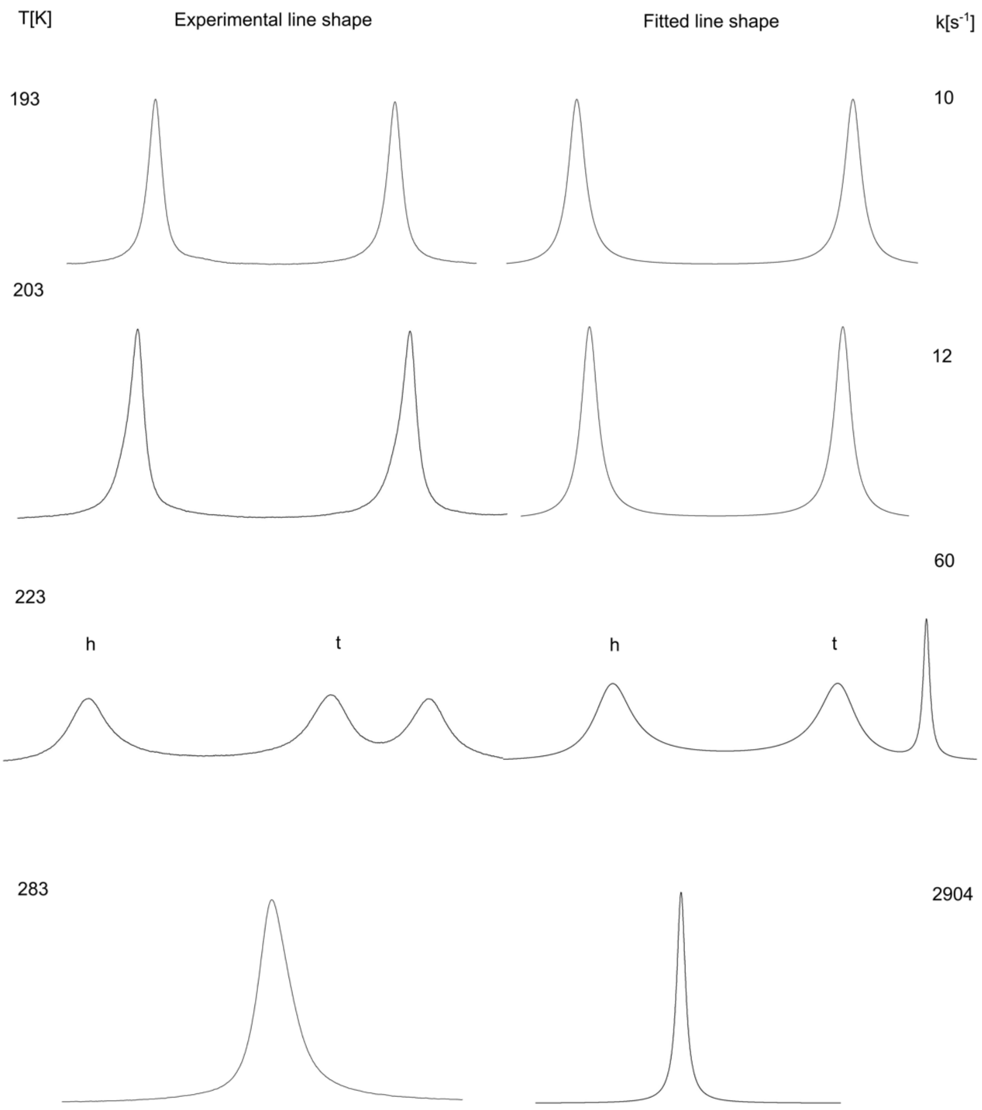

Fig. S-15 Complete line shape fitting results for resorcinol protons H_h and H_t, which were used for the Eyring plot of **2**.

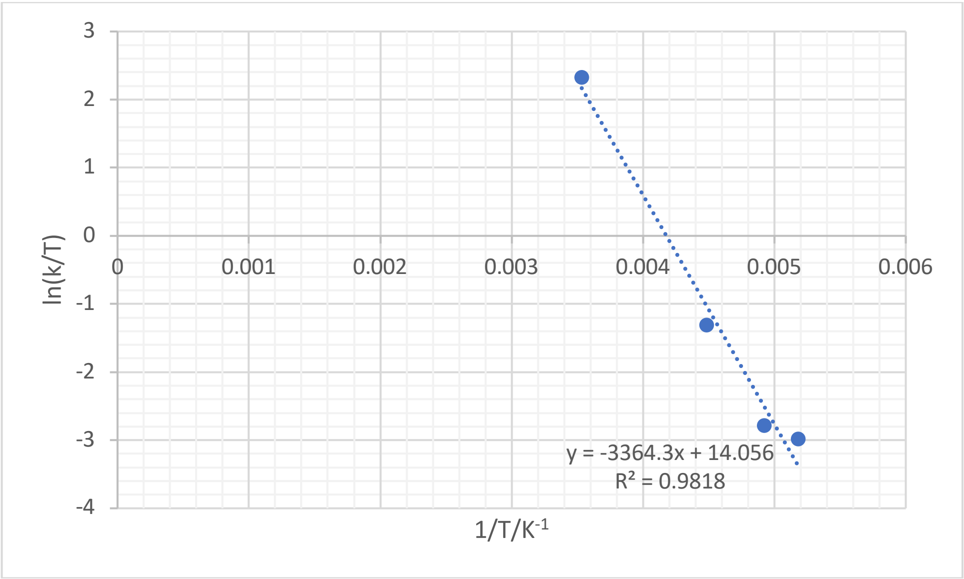

Fig. S-16 Eyring plot of **2**.

2D NMR experiments

Resorcinarene 1

The $^{1}H,^{1}H$ ROESY spectrum measured at 193 K showed the existence of strong negative ROE cross-peak between resorcinol upper rim proton H_h and methoxy proton H_g and a weaker signal between the corresponding ArH_t and H_s in the second resorcinol ring system. Strong ROEs were observed between resorcinol ArH_h and ethoxy protons that were preliminarily assigned as arising from H_e and H_f. In addition, methine protons H_j and H_v appeared as separate signals with positive exchange cross-peaks.

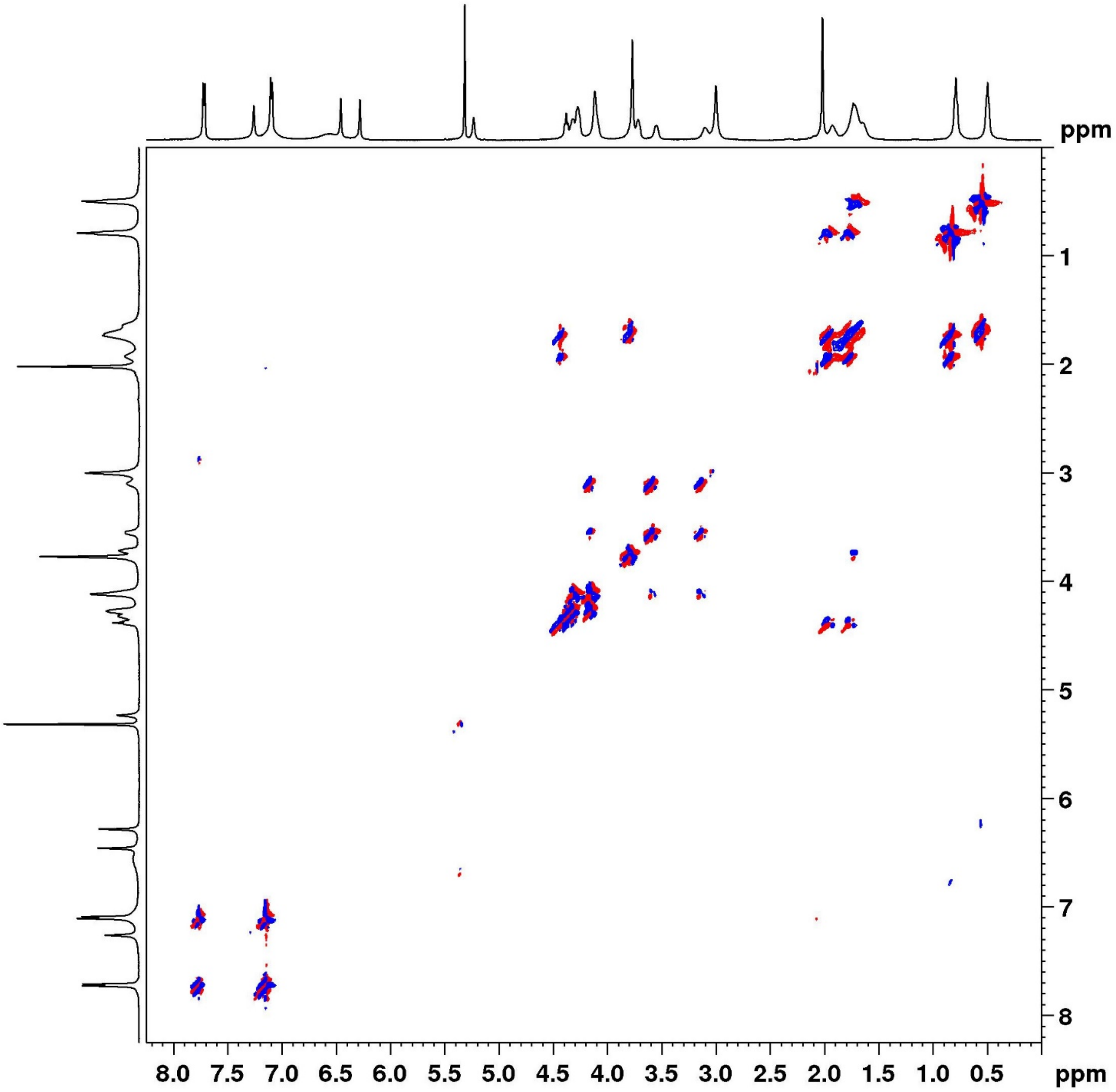

Fig. S-17 ^{1}H-^{1}H DQF-COSY NMR spectrum of **1** in CD_2Cl_2 (-75.5°C, 500 MHz).

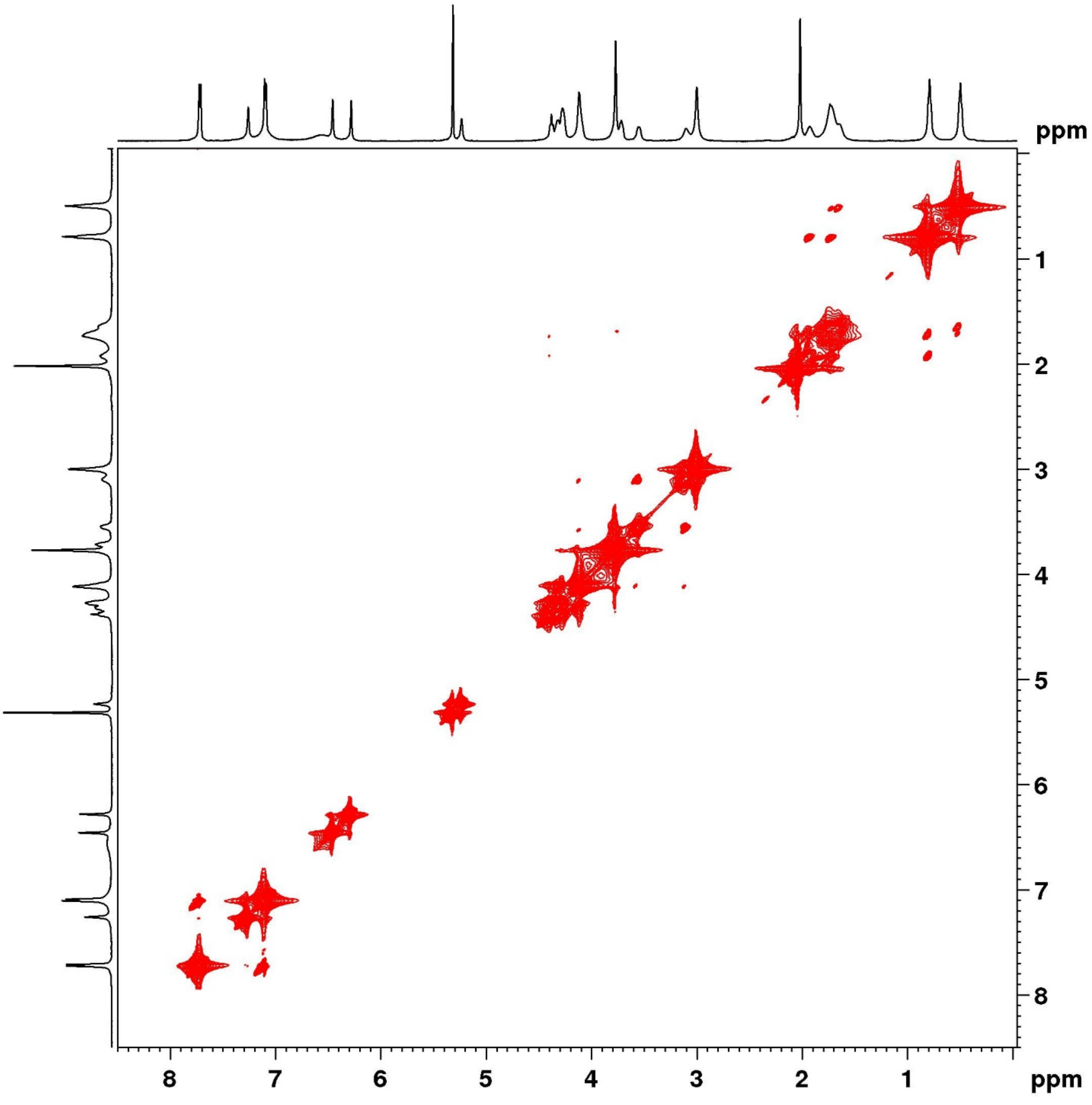

Fig. S-18 ^{1}H-^{1}H g-COSY NMR spectrum of **1** in CD_2Cl_2 (-75.5°C, 500 MHz).

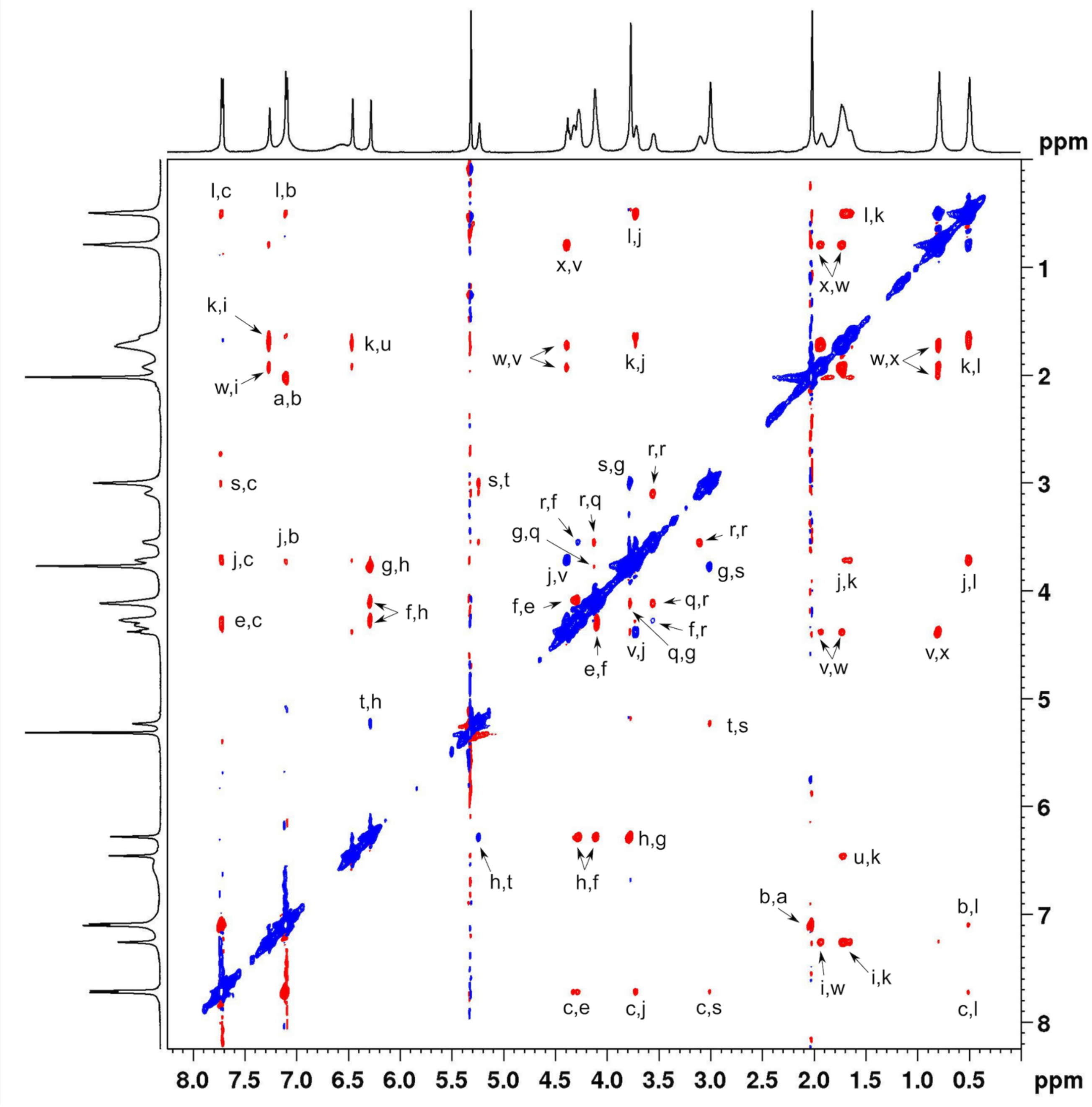

Fig. S-19 ^{1}H-^{1}H ROESY 2D NMR spectrum of **1** in CD_2Cl_2 (-75.5°C, 500 MHz).

Resorcinarene 2

The CH_2 protons H_w and H_k showed a ROE correlation to the multiplet resonating at 7.13 ppm, which indicates that the second lower rim aromatic H_u is overlapping with the signals of tolyl ring protons H_b, H_n, and H_o. In addition to ROE correlations between aryl and methoxy protons, positive exchange cross-peaks were observed between methoxy protons H_g and H_s, and upper rim protons H_h and H_t, respectively.

The OCH_2 protons were assigned to each spin system based on observed COSY correlations between H_f and H_e, as well as, between two pairs of diastereotopic signals arising from H_r protons alpha to resorcinol ring and H_q protons alpha to benzoyloxy group. In addition, ROE correlations from the H_q to benzylic H_p indicated that they belong to the same podand arm.

Unfortunately, the overlap of tolyl protons H_b, H_n and H_o, with lower rim H_u did not allow unambiguous assignment of ROE correlations from this pattern to benzylic protons H_d and H_p, and methoxy groups H_g and H_s. The cross-peak to H_g, however, most likely arises from the vertical aryl ring H_o since this is agreement with most of the computational models.

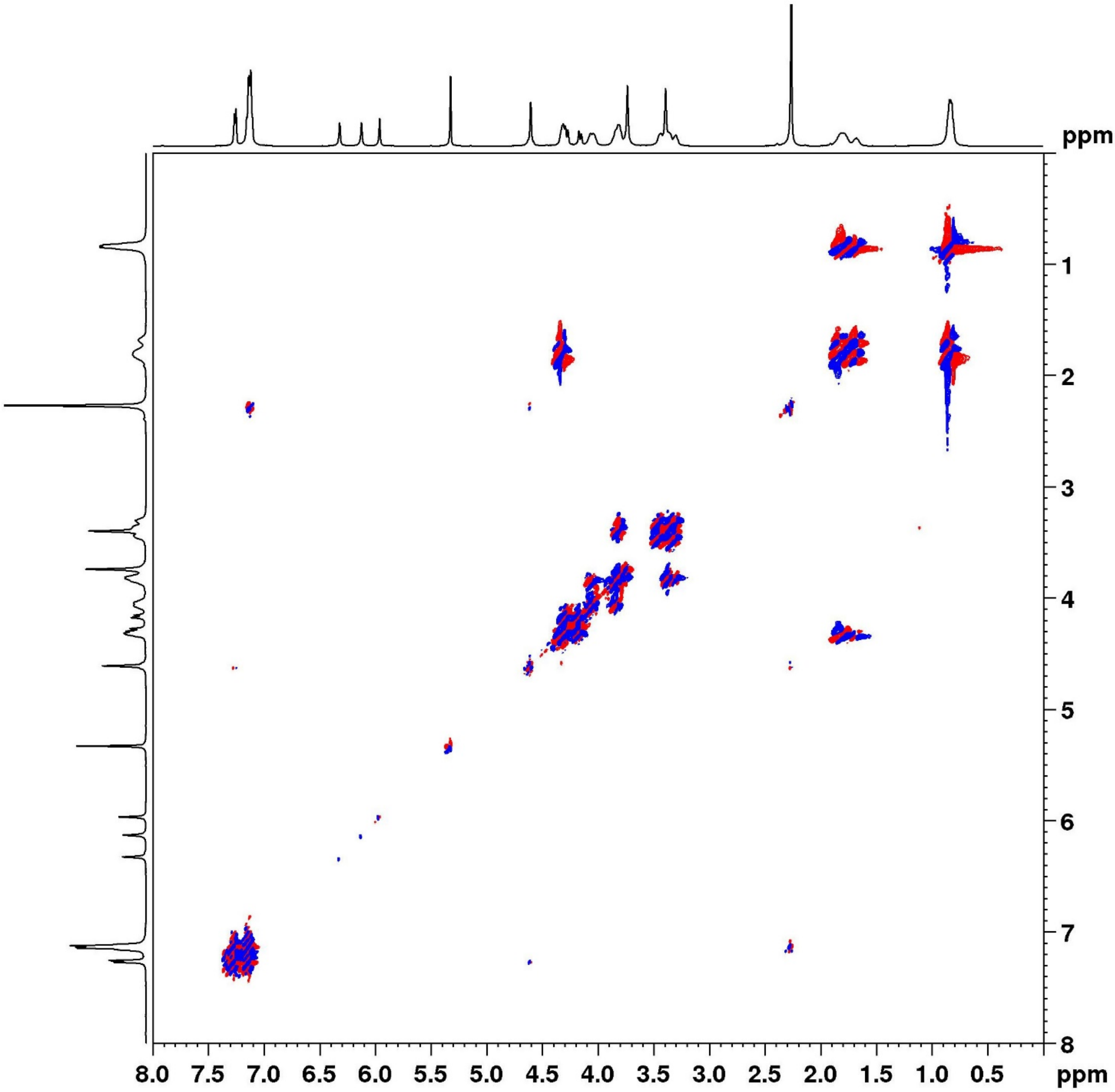

Fig. S-20 ^{1}H-^{1}H DQF-COSY NMR spectrum of **2** in CD_2Cl_2 (-75.5°C, 500 MHz).

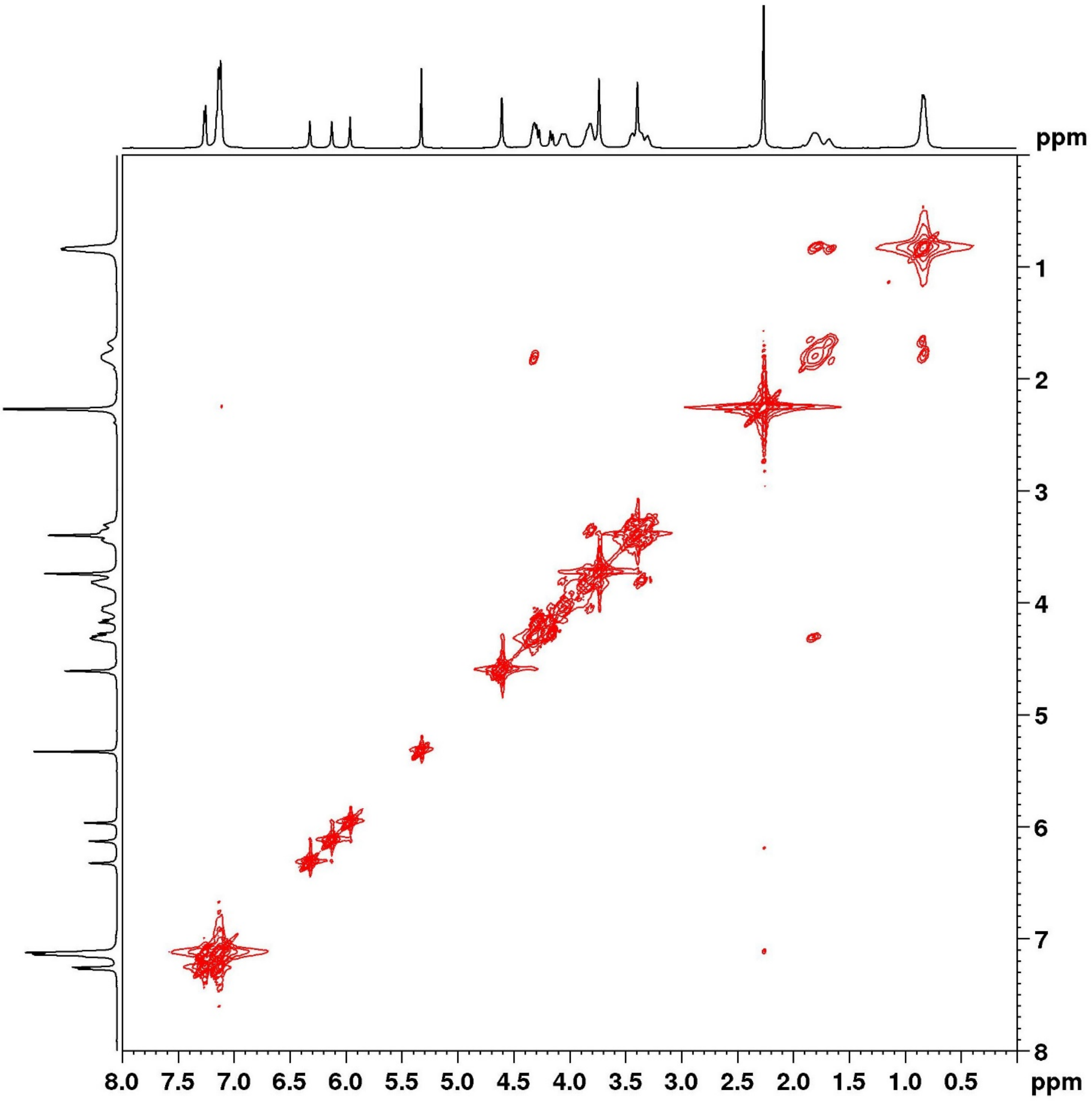

Fig. S-21 ^{1}H-^{1}H gCOSY NMR spectrum of **2** in CD_2Cl_2 (-75.5°C, 500 MHz).

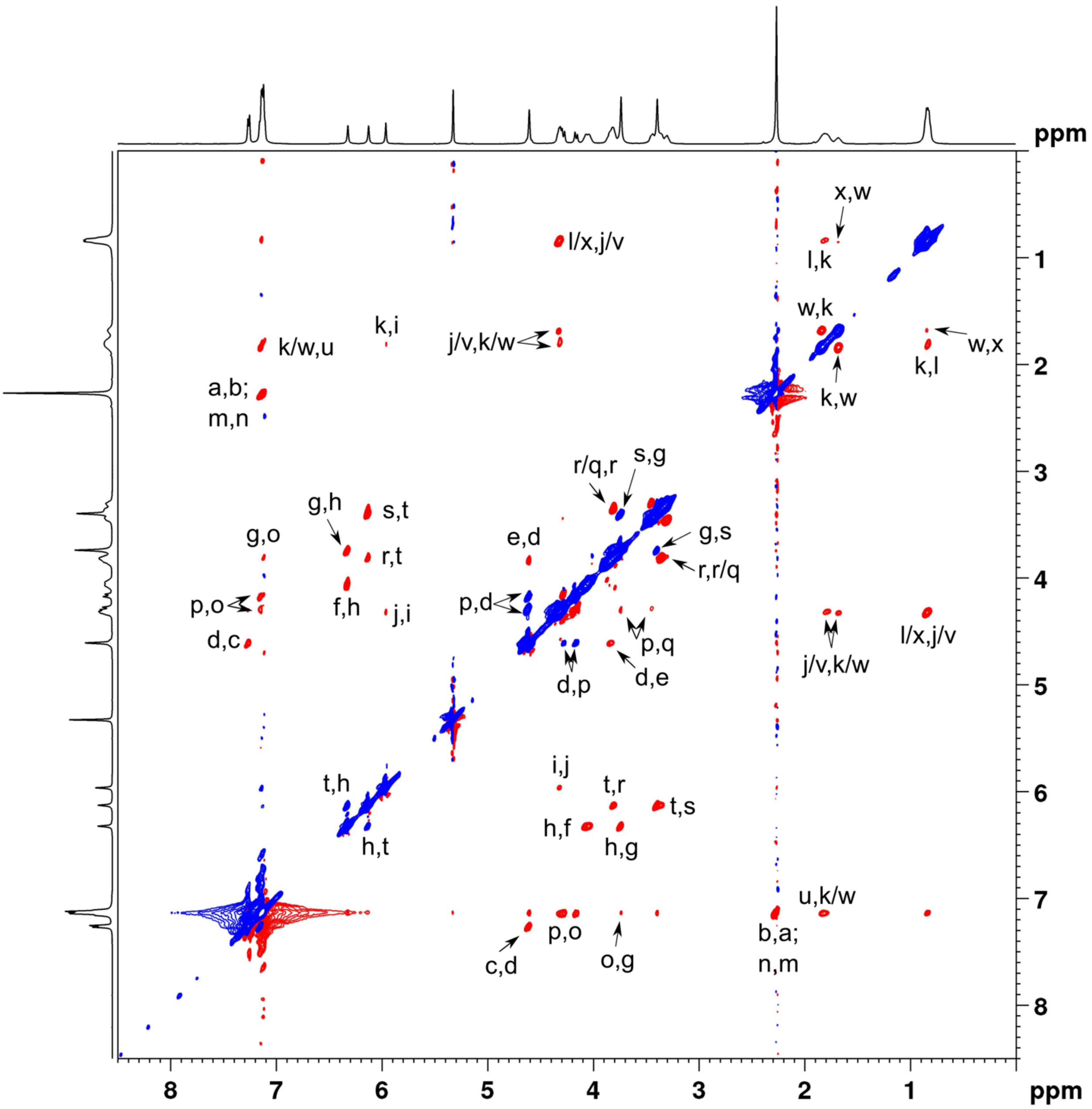

Fig. S-22 ^{1}H-^{1}H ROESY 2D NMR spectrum of **2** in CD_2Cl_2 (-75.5°C, 500 MHz).

X-ray crystallography

Table S1. Crystal data and collection parameters.

	**1**	**2**
Formula	$C_{76}H_{88}O_{20}S_4$	$C_{80}H_{96}O_{12}$
Crystallization solvent	ethyl acetate / hexane	ethanol
Crystal shape	block	block
Crystal colour	colourless	colourless
M/gmol^{-1}	1449.70	1249.56
Crystal system	Triclinic	Triclinic
Space group	P-1	P-1
a/Å	12.5704(5)	16.7219(13)
b/Å	15.7603(6)	19.1372(15)
c/Å	20.0036(6)	24.8269(14)
α/º	77.837(3)	94.647(5)
β/º	74.532(3)	108.745(6)
γ/º	75.713(3)	107.714(7)
V/Å^3	3656.5(2)	7024.6(9)
Z	2	4
ρ_{calc}/g cm^{-3}	1.317	1.182
Meas. reflns	26264	40058
Indep. reflns	16695	29287
T/K	123	120
R_{int}	0.0287	0.0342
R_1 [I> 2σ(I)]	0.0511	0.0700
wR_2 [I> 2σ(I)]	0.1284	0.1496
GooF	1.033	1.027

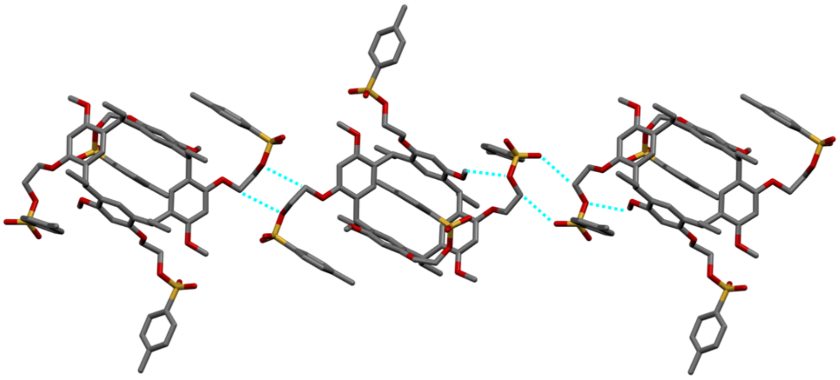

Fig. S-23 Crystal packing of **1** showing weak hydrogen bonds between horizontal sidearms. Hydrogen atoms have been omitted for clarity.

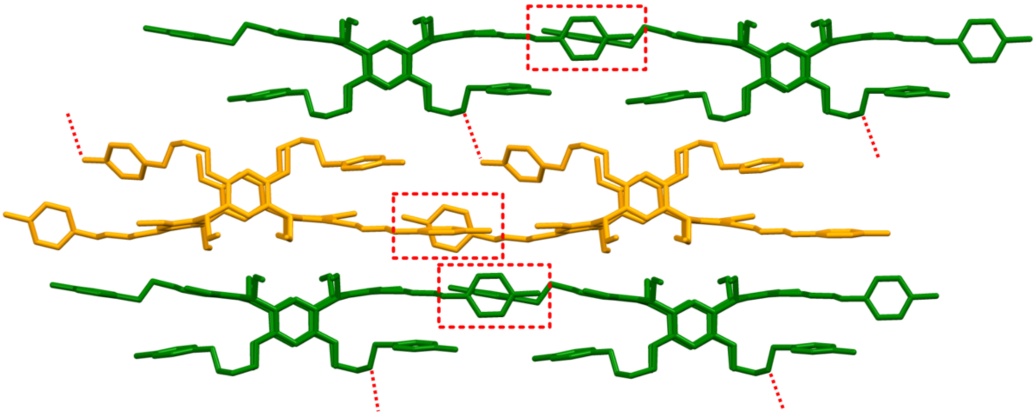

Fig. S-24 Crystal packing of **2** showing edge-to-face π··· π interactions between horizontal sidearms inside red boxes, and weak hydrogen bonds with dashed lines.

Computational results

DFT optimization of podands **1** and **2** was performed with two popular functionals B3LYP-D3 and PBE0-D3. The results for podand **1** were very similar with both functionals (Table S-2). The optimization for podand **2** gave some structural differences (Fig. S-24) and different values for relative energies (Table S-3) depending on the functional. However, the order and qualitative trend in energies remained the same.

Table S2. Relative energies of the computational structures for podand **1** (kJ/mol) obtained with molecular mechanics using OPLS3e force field, and two popular DFT functionals B3LYP-D3 and PBE0-D3 with 6-31G** basis set and PCM solvent model.

	OPLS3e	B3LYP-D3	PBE0-D3
**1**-I	0	0	0
**1**-II	1.12	5.72	5.19
**1**-III	2.26	10.45	7.87
**1**-IV	6.10	20.46	17.43
**1**-V	14.80	32.30	28.16

Table S3. Relative energies of the computational structures for podand **2** (kJ/mol) obtained with molecular mechanics using OPLS3e force field, and two popular DFT functionals B3LYP-D3 and PBE0-D3 with 6-31G** basis set and PCM solvent model.

	OPLS3e	B3LYP-D3	PBE0-D3
**2**-I	0	0	0
**2**-II	3.81	16.09	8.96
**2**-III	4.79	14.43	8.27
**2**-IV	7.29	50.00	30.74
**2**-V	11.08	6.10	1.80

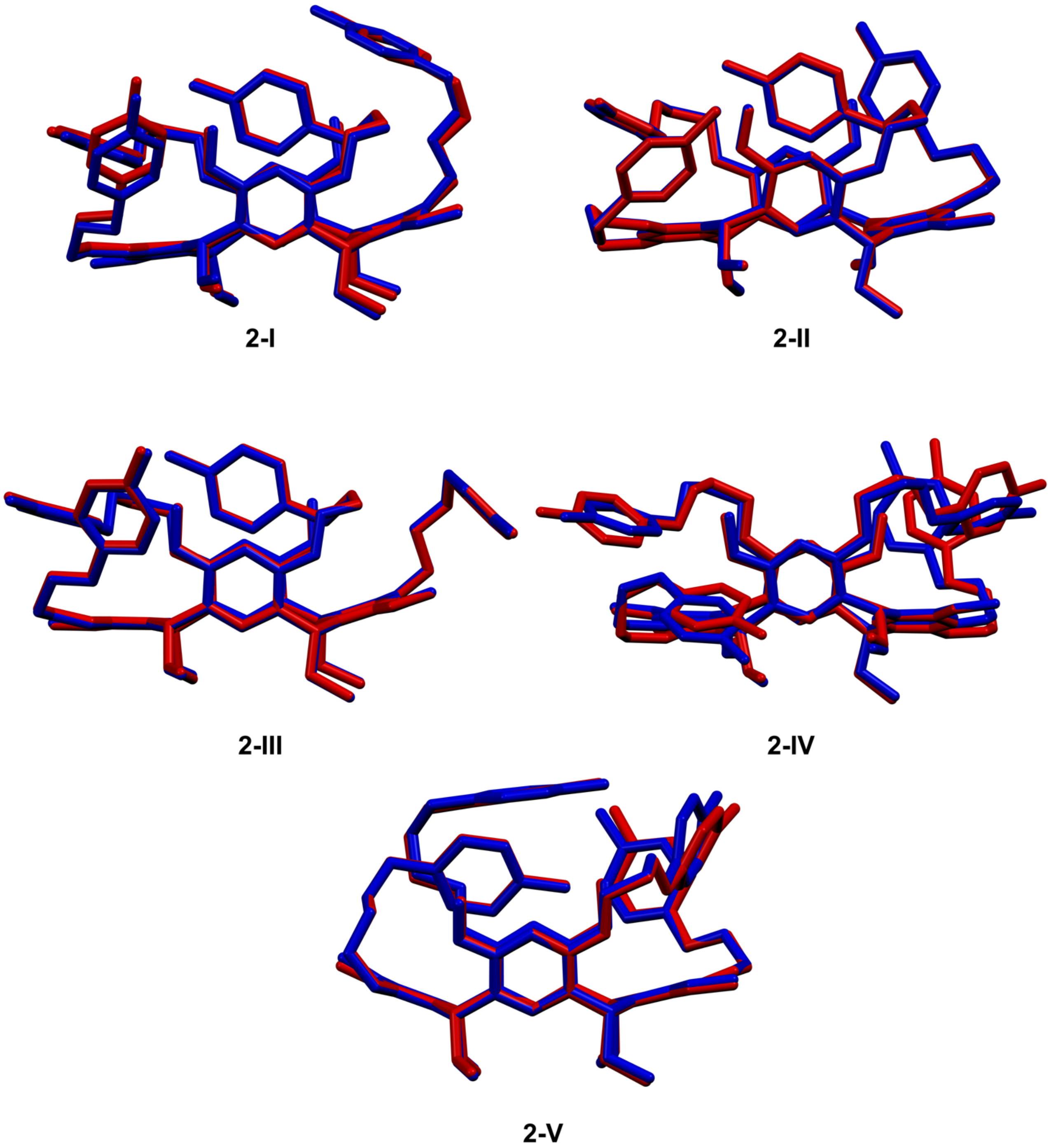

Fig. S-25 Overlay of B3LYP-D3 (blue) and PBE0-D3 (red) optimized structures of **2**.
